# Cambrian suspension-feeding lobopodians and the early radiation of panarthropods

**DOI:** 10.1186/s12862-016-0858-y

**Published:** 2017-01-31

**Authors:** Jean-Bernard Caron, Cédric Aria

**Affiliations:** 1 Department of Natural History (Palaeobiology Section), Royal Ontario Museum, Toronto, Ontario Canada; 2 Department of Ecology and Evolutionary Biology, University of Toronto, Toronto, Ontario Canada; 3 Department of Earth Sciences, University of Toronto, Toronto, Ontario Canada

**Keywords:** Onychophora, Velvet worm, Cambrian evolutionary radiation, Stem-group, Disparity

## Abstract

**Background:**

Arthropoda, Tardigrada and Onychophora evolved from lobopodians, a paraphyletic group of disparate Palaeozoic vermiform animals with soft legs. Although the morphological diversity that this group encompasses likely illustrates the importance of niche diversification in the early radiation of panarthropods, the ecology of lobopodians remains poorly characterized.

**Results:**

Here we describe a new luolishaniid taxon from the middle Cambrian Burgess Shale (Walcott Quarry) in British Columbia, Canada, whose specialized morphology epitomizes the suspension-feeding ecology of this clade, and is convergent with some modern marine animals, such as caprellid crustaceans. This species possesses two long pairs and four shorter pairs of elongate spinose lobopods at the front, each bearing two slender claws, and three pairs of stout lobopods bearing single, strong, hook-like anterior-facing claws at the back. The trunk is remarkably bare, widening rearwards, and, at the front, extends beyond the first pair of lobopods into a small “head” bearing a pair of visual organs and a short proboscis with numerous teeth. Based on a critical reappraisal of character coding in lobopodians and using Bayesian and parsimony-based tree searches, two alternative scenarios for early panarthropod evolution are retrieved. In both cases, hallucigeniids and luolishaniids are found to be extinct radiative stem group panarthropods, in contrast to previous analyses supporting a position of hallucigeniids as part of total-group Onychophora. Our Bayesian topology finds luolishaniids and hallucigeniids to form two successive clades at the base of Panarthropoda. Disparity analyses suggest that luolishaniids, hallucigeniids and total-group Onychophora each occupy a distinct region of morphospace.

**Conclusions:**

Hallucigeniids and luolishaniids were comparably diverse and successful, representing two major lobopodian clades in the early Palaeozoic, and both evolved body plans adapted to different forms of suspension feeding. A Bayesian approach to cladistics supports the view that a semi-sessile, suspension-feeding lifestyle characterized the origin and rise of Panarthropoda from cycloneuralian body plans.

**Electronic supplementary material:**

The online version of this article (doi:10.1186/s12862-016-0858-y) contains supplementary material, which is available to authorized users.

## Background

Lobopodians are known from more than 30 species preserved in various Palaeozoic fossil Lagerstätten across the globe [1], with the best preserved material coming from Cambrian Burgess Shale-type deposits in China and Canada [2–7]. Lobopodians were common members of many Cambrian benthic communities globally; many skeletal elements also occur as isolated carbonaceous and phosphatic microfossils in many other types of deposits [8]. Interpretations of their ecological roles, however, have long remained marginal to studies of their morphology. While the discovery of different lobopodian body plans suggest different lifestyles [7], it is only recently that an attempt has been made to quantify the degrees of morphological and ecological disparity displayed by these organisms [9]. This work has led to the suggestion that the luolishaniids, a group distinguished notably by pronounced antero-posterior limb differentiation [7, 9], was significantly more disparate than extant onychophorans and occupied a unique morpho-functional niche among their relatives [9]. Following recent research on the morphology of *Hallucigenia* [3], this study [9] primarily relied on the armoured *Collinsium* [9] and *Luolishania* [7] to hypothesize that luolishaniids were stem-group onychophorans characterized by a specialization in suspension feeding. This view of the early evolution of panarthropods was in need of both additional morphological evidence related to suspension feeding in these fossils and of a cross-validation of the phylogenetic results put forward by the authors [9], who, notably, supported tardigrades as a sister group to a total-group Euarthropoda (forming a Tactopoda clade).

The new luolishaniid lobopodian described here from the Burgess Shale (Walcott Quarry) was first reported along with two other forms at a conference in 2001 [10]. One of these other forms is the “Collins monster” [11–14] from the Burgess Shale’s Tulip Beds (“Undet Lobopodian TB-A” in [15]), a species that, despite the lack of formal description and details about its morphology, has repeatedly been discussed and incorporated in a number of phylogenetic analyses, including those of Yang et al. [9]. The new species described here represents only the third lobopodian to be formally described from the Burgess Shale after *Hallucigenia sparsa* [4, 16] and *Aysheaia pedunculata* [17], which were first reported in 1911 [18]. With only two specimens discovered to date, it is also one of the rarest species from the Burgess Shale community.

## Methods

### Observations

The specimens were studied in a manner similar to other Burgess Shale specimens (e.g. [19]): they were prepared using light mechanical tools to remove matrix coating some body elements, observed using a stereomicroscope, and photographed under different lighting conditions, including interference lighting (see Additional files 1, 2, 3, 4, 5, 6, 7, 8 and 9). The holotype specimen was also observed using a scanning electron microscope (FEI Quanta 200 Field Emission Gun), uncoated and operated under low or high vacuum conditions between 10.0 kV and 20.0 kV, to obtain secondary electron or backscattered images and elemental maps. Elemental maps were collected using an EDAX energy dispersive spectroscopy (EDS) X-ray detector under low vacuum (70 KPa). *Phylogenetic treatment*. Cladograms were produced using MrBayes v. 3.2.6 [20] and PAUP* v.4.0a150 [21] based on a dataset of 59 characters and 38 taxa. The Bayesian analyses used the Mkv method recommended by Lewis for morphological data [22] (i.e., with the assumption that all observed characters are variable with equal transition rates between states). Trees were generated during two parallel runs of 10,000,000 generations (four chains) with a tree sampled every 1000 generation and burn-in of 20%. Estimation of variation in among-character rates was set to follow a gamma distribution. The parsimony analysis consisted in a heuristic search with tree bisection reconnection, for which we constrained a limit of 10 trees of scores ≥ 1 for each of the 1000 replicates. All characters remained unweighted and unordered in all analyses and inapplicable entries were treated as uncertainties (see Additional files 10, 11 and 12 for further methodological details, and datasets). *Disparity analysis*. The morphospace analysis follows the method we used in a previous study [23]. Sums of ranges were jackknifed for each group using 5000 iterations, from which permutation statistics were calculated as well as their corresponding *p*-values (following, e.g., [24]). The R package *hypervolume* was used to generate volume data based on kernel density estimates of the Euclidean coordinates on the first four principal coordinate analysis (PCoA) axes (the four significant factors of variation under a Broken Stick model), and overlap was tested using the Sorensen-Dice index [25]; this method to test the inclusion of points in a specific hypervolume is also provided with the *hypervolume* package. Additional R packages, namely, *cluster*, *scatterplot3d*, *vcd*, and *vioplot*, were used to produce graphics. The tree search and morphospace use different datasets (see Additional file 10 for further information).

## Results

### Systematic palaeontology

Superphylum Panarthropoda Nielsen, 1995

Family Hallucigeniidae Conway Morris, 1977

**Type genus and species**. *Hallucigenia sparsa* Walcott 1911 [18].

**Diagnosis** (revised from references [16, 26]): Lobopodian panarthropods characterized by long, tubular and smooth lobopods, but commonly adorned with well-developed pairs of plates or spines dorso-laterally on the trunk. Members of this family characteristically exhibit a small ovoid or large bulbous head followed, respectively, by an elongate or short “neck” bearing two pairs of non-annulated, flexible anterior tentacle-like limbs that are thinner than trunk lobopods and do not bear any spinules or claws.

### Included taxa

*H. sparsa* and *H. hongmeia*, from the early Cambrian Stage 4 Guanshan fossil Lagerstätte [27], *Cardiodictyon catenulum* [26, 28] and *H. fortis* [12, 26] from the early Cambrian (Stage 3) Chengjiang Biota (Maotianshan Shale Member, Yu’anshan Formation), and *Carbotubulus waloszeki* from the Middle Pennsylvanian Carboniferous of Illinois [29].

### Remark

The inclusion of *Microdictyon sinicum* [30] (and other related species from various localities—e.g. [31]) within the hallucigeniidae is supported by our Bayesian topology but not by parsimony (see below). *Microdictyon* is otherwise found to conform to the hallucigeniid body plan by our disparity analysis. Since tentacles are absent in this taxon, we keep it outside Hallucigeniidae, but the “elongate” condition of its head similar to *H. sparsa* and *Carbotubulus* may require further discussion and the creation of a more inclusive hallucigeniid clade.

Family Luolishaniidae Hou & Bergström, 1995

**Type genus and species**. *Luolishania longicruris* Hou and Chen, 1989 [32].

**Diagnosis** (emended from [9]): Lobopodian panarthropods with anteriormost five or six lobopod pairs adorned with double rows of 20–30 spinules arranged in a chevron-shaped pattern along the ventral side; body tagmatization includes both anteriorward and posteriorward gradual reduction in lobopod interspace, with posterior tagmatization always involving more than two limb pairs; when dorso-lateral spines are present, they are complemented by at least a third, median row of spines [see also Additional file 10].

### Included taxa

*Ovatiovermis* (infra), *Collinsium ciliosum* [9] from the early Cambrian (Stage 3) of China, an unnamed species from the early Cambrian (Stage 4) of Australia [33], *Acinocricus stichus* [12, 34] from the middle Cambrian (Stage 5) of the USA.

### Remark

The “Collins monster” [11, 12] and another unpublished form [14] from the Burgess Shale are currently being described by the authors and were not included in this paper; their affinities within luolishaniids, e.g. [9], require further investigation. In both Bayesian and parsimony topologies, *Facivermis yunnanicus* Hou and Chen is found to be part of the luolishaniid clade, owing to the presence in this taxon of the diagnostic double-row of spinules on its anterior—and only—limbs. The lack of posterior limbs could be either plesio- or autapomorphic; as it stands, *Facivermis* may be later included in a more inclusive luolishaniid clade.

*Ovatiovermis cribratus* gen. et sp. nov.

LSID urn:lsid:zoobank.org:act:DE92964E-9843-469E-ADBD-8A34374AF286

LSID urn:lsid:zoobank.org:act:6331FB8C-DCCB-4782-AAC2-57C3281D0639

### Etymology

*Ovatiovermis* is from the Latin *ovatio* (ovation) and *vermis* (worm) owing to the inferred upward-reaching, limb-waving posture of these animals; *cribratus* is from the Latin *cribrare*, to sieve.

### Type material

**Fig. 1:**
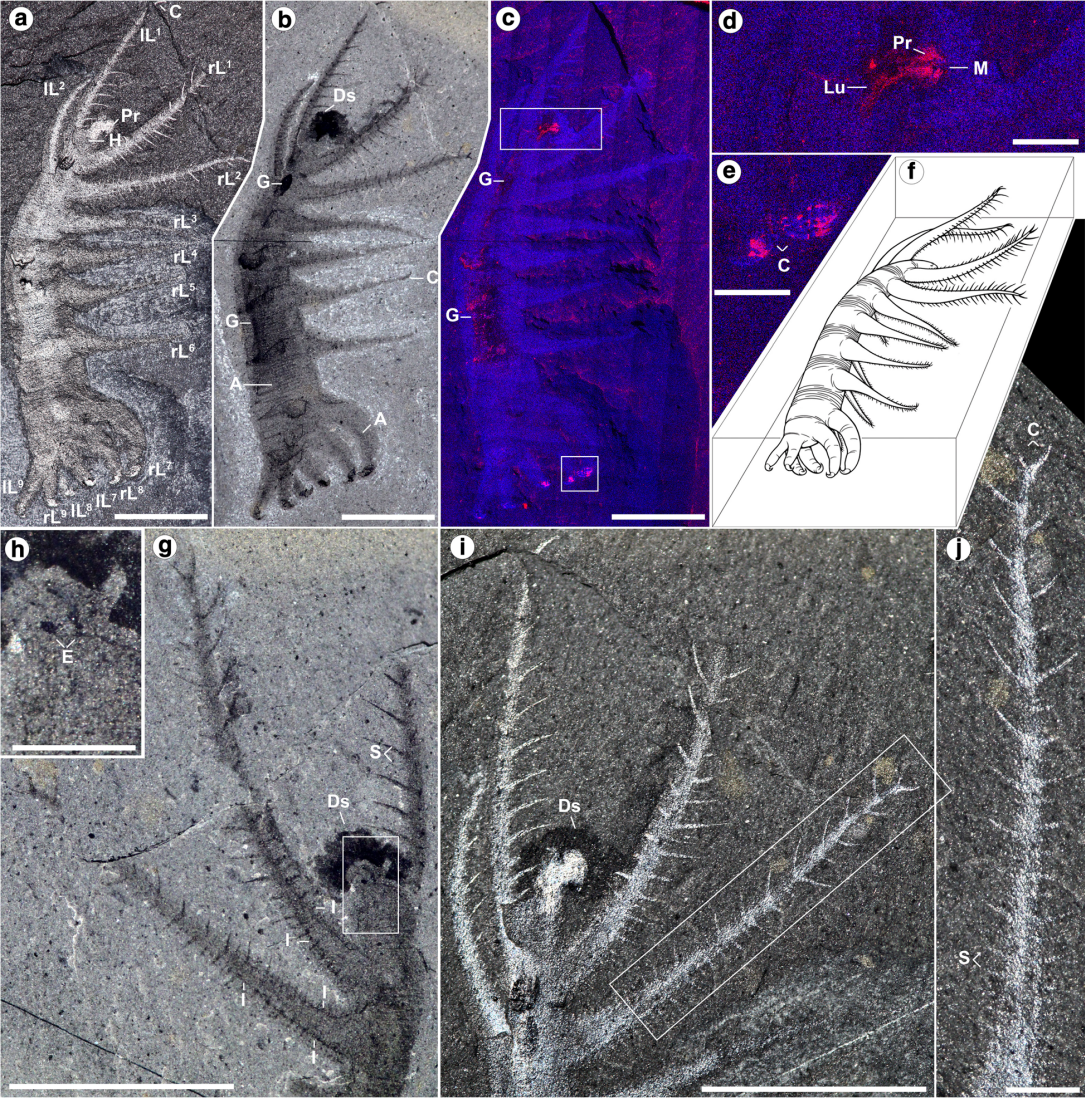
*Ovatiovermis cribratus* from the Burgess Shale, Royal Ontario Museum (ROM) 52707: (**a**–**e**, **i**, **j**) part, (**g**, **h**) counterpart, (**f**) reconstructed death pose. Close-ups indicated by white rectangles. **a**, **b** full specimen under direct (**a**) and polarized (**b**) lighting conditions. **c**–**e** superposed elemental maps of carbon (*red*) and calcium (*purple*) before preparation of the 8th left lobopod (lL^8^—see a). The lighter colours represent higher concentrations of elements: parts of the gut, proboscis, pharyngeal area and claws are preserved in carbon whereas the rest of the body is preserved in calcium (see also Additional file 6). **g**–**j** details of the anterior part of the body showing internal organs in lobopods (**g**), pair of visual organs (**g**, **h**), spinules and bifid claws (**g**, **i**, **j**). Digital single-lens reflex (DSLR) images taken using direct light (**a**, **i**, **j**) and cross-polarized light (**b**, **g**, **h**), all under dry conditions except (**b**, **g** and **h**). A, annulations; C, claw; Ds, dark stain; E, “eye” (visual organ); G, gut; H, head; I, internal organ; L, lobopod (l, left; r, right; ^x,y^, lobopod position); Lu; foregut lumen; M, mouth; Pr, proboscis; S, spinules. Scale bars: 5 mm (**a**–**c**, **g**, **i**), 1 mm (**e**, **d**, **h**, **j**)

**Fig. 2:**
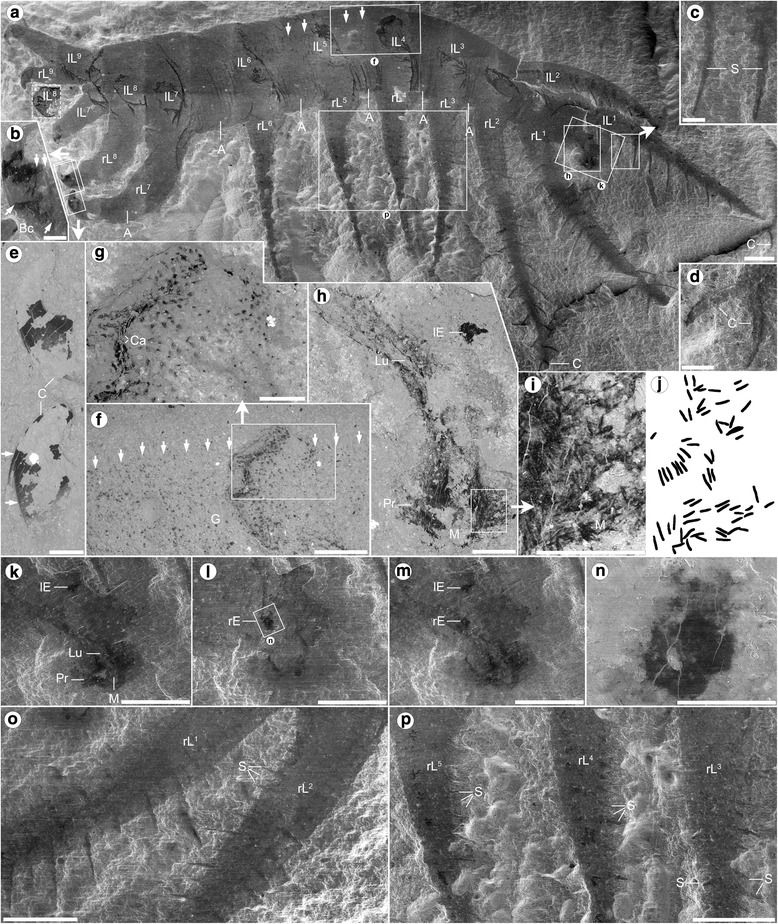
*Ovatiovermis cribratus* from the Burgess Shale, Royal Ontario Museum (ROM) 52707: scanning electron microscopy images in secondary electron mode (**a**–**d**, **k**–**m**, **o**, **p**) or backscatter mode (**e**–**i**, **n**). Close-ups indicated by *white rectangles* (*solid lines*) and arrows. **a** Full specimen (the 8th left lobopod, lL^8^—*dashed white rectangle*—photographed after preparation) and details of a posterior claw (**b**), spinule (**c**), and bifid anterior claw (**d**). **e** posterior single claws showing one possible stacked element (*arrows*). **f**, **g** black, carbonaceous remnants of the gut (dorsal side of the gut defined by vertical arrows; compare with (**a**)), particularly visible along body folds created by the compression of the opposite lobopod pair. **h** detail of the heard showing proboscis, pharyngeal area and one visual organ. (**i**–**j**) tooth-like structures along the proboscis (dark elements in drawing **j**). **k**–**m** detail of the “head” showing the left and right visual organs preserved on the part (**k**) and counterpart (**l**) respectively; (**m**) images of part and counterpart superposed using Calculation mode in Adobe Photoshop CS6 showing both visual organs together. **n** detail of the right visual organ. **o**, **p** detail of the anterior lobopods on the counterpart and detail of posterior lobopods (rL^3–5^) on the part showing spinules. A, annulations; Bc, basal unit of the claw; C, claw; Ca, compression artifact; Ds, dark stain; E, “eye” (visual organ); G, gut; H, head; I, internal organ; L, lobopod (l, left; r, right; ^x,y^, lobopod position); Lu; foregut lumen; M, mouth; Pr, proboscis; S, spinules; T, teeth. Scale bars: 1 mm (**a**, **o**, **p**), 0.5 mm (**f**, **k**–**m**), 0.2 mm (**b**–**e**, **g**, **h**), 0.1 mm (**i**, **j**, **n**)

Holotype ROM (Royal Ontario Museum) 52707, part (Figs. 1a–e, i–j; 2a–c, e–k, p; Additional files 1, 2, 4, 5, 6 and 7) and partial counterpart (Figs. 1h, g; 2d, l, n, o; Additional files 3 and 8), Paratype ROM 64006 (Additional file 9).

### Locality and stratigraphy

Walcott Quarry on Fossil Ridge (Yoho National Park, British Columbia), Walcott Quarry Shale Member of the “Burgess Shale” Formation [35]. The holotype specimen (ROM 52707) was collected from bed assemblage −120 (about 1.2 m below the base of the original Walcott Quarry floor), which is distinct from other bed assemblages in that it has particularly well-preserved specimens [36] across a range of taxa (N = 92 species) [37]. The paratype specimen (ROM 64006) comes from talus material originating from the Walcott Quarry.

### Diagnosis

*Ovatiovermis* differs from other luolishaniid lobopodians in possessing an unadorned trunk with the first two anteriormost limb pairs 1.5 times longer than lobopods of the midsection and a trunk width doubling posteriorly, ending in three pairs of increasingly shorter and very stout unadorned lobopods, each terminating in a single strongly sclerotized curved claw facing anteromesially.

### Description

**Fig. 3:**
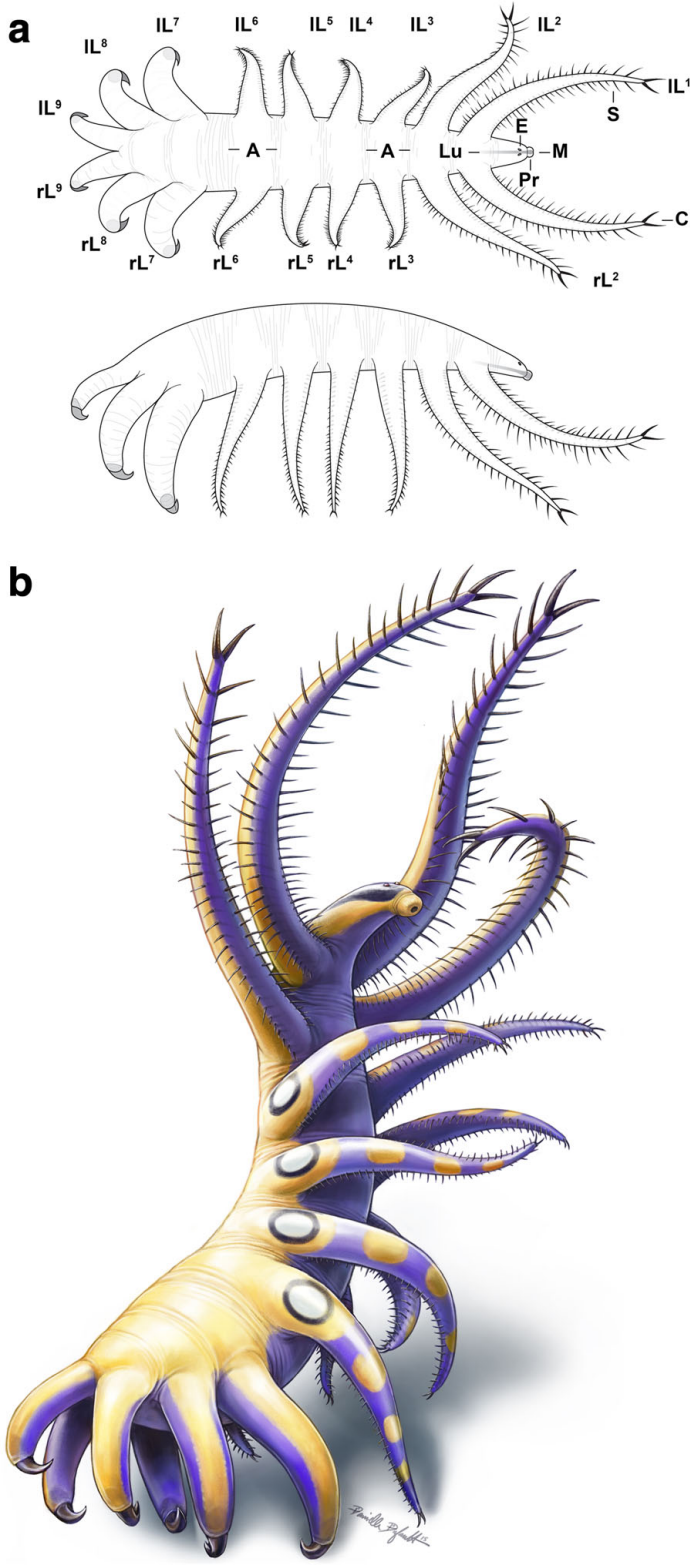
*Ovatiovermis cribratus* from the Burgess Shale, anatomical drawings of the dorsal and lateral views (**a**) and artistic representation (**b**). Drawings by Danielle Dufault. (See also electronic Additional files 13 and 14)

The holotype specimen (ROM 52707) is compressed obliquely with a dorso-ventrally upturned front end (Fig. 1f), and the trunk measures c. 18 mm in length (c. 30 mm with first and last pairs of lobopods fully extended). The paratype specimen (ROM 64006) is also compressed obliquely, including the front end, and the trunk is smaller (c. 12 mm in length), but otherwise the specimen is generally less well preserved compared to the holotype specimen (Additional file 9), and therefore, the description below is based on the holotype only. All lobopods are visible in full or in part, except that the left lobopods 3 to 6 are concealed beneath the body, as suggested by elliptical integumental folds above the leg insertions (e.g. Figs. 1b and 2a, f, g; Additional files 1, 2, 4 and 7) in a manner similar to that seen in *Aysheaia* (e.g. plate 10 and figure 62 in [17]). The trunk is widest at the level of the third last pair of lobopods (c. 3.5 mm) and narrowest before the first pair of lobopods (c. 1.5 mm) at the front (Figs. 1a, 2a and 3)

. It shows a variable number of fine epidermal annuli (c. 10 annuli/mm) between limb pairs (Fig. 2a; Additional files 2, 4 and 7). The section before the first limb pairs, interpreted here as the “head,” is small (c. 10% of total trunk length) and bears a pair of dorsal ovoid structures about 100 μm in length (Figs. 1h and 2h, k–n) interpreted as visual organs, likely ocellar—no evidence of more complex eye structures like in *Luolishania* and other forms [6] is preserved. A darker axial structure rich in carbon (Fig. 1c–d; Additional file 6) starting from the distal end of the head and running to at least two-thirds of the way through the trunk is interpreted as a short bulbous proboscis with a terminal mouth followed by a foregut with a distinct lumen (Fig. 2h). A dark stain around the head (Fig. 1b, g–i; Additional files 1, 2 and 3) probably represents decay fluids of internal tissues seeping out of the mouth. The proboscis bears dozens of tooth-like elements (Fig. 2i–j), each c. 20 μm in length, with no evidence of such elements present in the foregut. Small carbonaceous spots of various shapes and sizes further back along the trunk along a confined axial zone probably represent gut remains. These are more obvious along folded areas (e.g. Fig. 2f, g), especially above the insertion points of the opposite limb pairs. Like in *Luolishania* [7]*,* a pair of conspicuous darker strips c. 0.2 mm in width is evident within the first two pairs of lobopods (Fig. 1g; Additional files 3 and 5). These seem to be linked to the basis of the spinules and possibly represent internal structures such as neural tissues or blood vessels. Three types of lobopods can be identified (Figs. 1 and 2). The first two pairs of lobopods are the longest (c. 9.7 mm and c. 9 mm respectively), followed by the 3rd to 6th pairs (average 5.6 mm), and 7th to 9th pairs (from c. 5.3 mm to c. 3.5 mm for the last pair). The first six pairs are elongate, broad at the base (c. 1.2 − 1.4 mm) and quickly narrowing towards the tip (the base of a narrow elongate claw), whereas the last three pairs have a wide base (decreasing from 1.5 to 1 mm at the back) and narrow only slightly towards the tip. The distance between pairs of lobopods seems constant between pairs 1 to 5, and increases between pairs 5 and 6 (c 1.2 mm), and 6 and 7 (c. 2 mm). The distance between each of the last three pairs of lobopods, however, is much smaller (c. 0.3 mm). Twenty to thirty pairs of spinules are present on the first two pairs of lobopods (Fig. 1i, j), varying in length and thickness, possibly due in part to variations in the angle of burial and minor rotations of the lobopods, the longest being c. 1 mm long and 0.1 mm thick. While spinules are present along the entire length of the lobopods, the longest spinules tend to be positioned distally. Spinules are much more diffuse and shorter along the 3rd to 6th pairs of lobopods, and there is no evidence of spinules on the last three pairs of lobopods. Spinules are inserted medially along the ventral surfaces of the lobopods (Fig. 2o), roughly 0.2 mm apart and arranged in a V (chevron) shape, forming a roughly 100° angle. A pair of elongate (c. 1 mm long and c. 0.1 mm thick) and slightly curved claws is present at the end of each of the first two pairs of lobopods (Figs. 1j and 2d), and possibly the following four pairs as well, although they are not well preserved. The last three pairs of lobopods have strongly curved (c. 150°) claws c. 1 mm in length (Figs. 1e and 2a, b, e). These claws have a broad base (Fig. 2a) which is poorly sclerotized compared to the tips (Fig. 2e), and might represent an internal support to the claw. The claws narrows to a smooth tip, and contain two stacked elements (Fig. 2e) similar to those in *Hallucigenia* [4]. Also similar to the last two pairs of claws in *Hallucigenia* [4] and *Collinsium* (Fig. 2c in [9]), the last three pairs of claws in *O. cribratus* point towards the front.

### Phylogenetic analysis

**Fig. 4:**
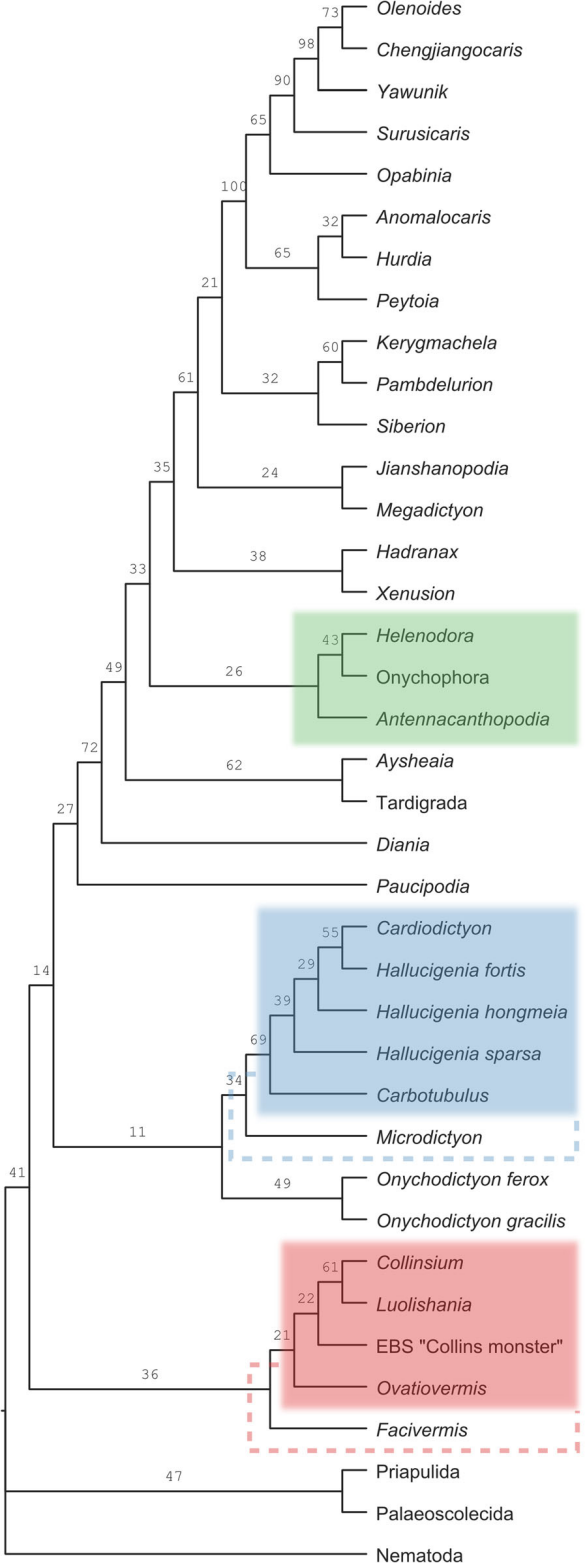
Consensus tree of a Bayesian phylogenetic analysis of our morphological matrix composed of 38 taxa and 59 characters (four runs, 10,000,000 generations, burn-in fraction: 0.20; see Nexus file, Additional file 9). Total-group onychophorans highlighted in *green*, hallucigeniids in *blue* and luolishaniids in *red. Microdictyon* and *Facivermis* are considered potentially included within hallucigeniids and luolishaniids, respectively, which is denoted by the *coloured dashed lines*. Numbers above branches represent posterior probabilities

Bayesian- and parsimony-based tree searches find two alternative scenarios for early panarthropod evolution (Fig. 4; Additional file 10: Figure S1) that have in common the close relationship and respective monophyly of hallucigeniids and luolishaniids, with Onychophora, Tardigrada and Arthropoda retrieved outside of these clades

The Bayesian consensus favours a basalmost monophyletic Luolishaniidae and a derived monophyletic Hallucigeniidae as sister-group to the remaining panarthropods, with tardigrades and onychophorans forming a grade to Arthropoda. The position of hallucigeniids, luolishaniids and *Onychodictyon* taxa relative to each other is not strongly supported, however, showing that basal relationships between “long-legged” forms (see [29]) is still difficult to resolve. In contrast, parsimony resolves onychophoran-like taxa as a basalmost grade, with tardigrades as sister group to a monophyletic Hallucigeniidae + Luolishaniidae, itself sister clade to “kerygmachelids” + Arthropoda—a Tactopoda configuration *sensu* [3]. The result under parsimony is strongly influenced by the polarization of integumental differentiations.

While the paraphyly of lobopodians was also found in previous studies [3, 4, 9], neither of the two topologies corroborates findings of a monophyletic “total onychophoran group” including hallucigeniids and luolishaniids as retrieved from a recent dataset ([3, 9]; see Additional file 10). The “cone-in-cone” sclerite, a character presented as critical in grouping hallucigeniids and onychophorans [3], is here optimized as plesiomorphic due to uncertain coding in most of the relevant taxa.

The grouping of *Aysheaia* with tardigrades is an illuminating new result for the origin and evolutionary significance of this group, as is its position relative to luolishaniids and hallucigeniids under parsimony. *Aysheaia* displays multiple juxtaposed claws at the tips of their lobopods that could constitute a possible apomorphy of the group. Modern tardigrades have lost large intermediate regions of their body axis [38], hampering otherwise a straightforward relationship with fossils based on external anatomy. Nonetheless, our study puts emphasis on typical tardigrade characters that, along with onychophoran ones (e.g. annulation, lobopods, ocular appendages), have shaped early panarthropod evolution, namely, the posterior body termination characterized by truncation, and forward-facing claws. These traits have been present throughout the stem Panarthropoda, with, as we discuss below, potentially important ecological implications. On the other hand, our phylogenetic results do not permit to conclude on whether the dorsal and ventral plates of heterotardigrades relate to either lobopodian sclerites or arthropod tergo-sternites [39]. Likewise, the presence of a ganglionic ventral nerve cord ([40], the only sound synapomorphy of Tactopoda in [9]), while indeed a strong argument to bring tardigrades closer to arthropods, is not incompatible with a reversal in onychophorans from a likelihood perspective. As a result, we think that the Tactopoda concept [41] is not well supported from a morphological point of view. Molecular analyses likewise disagree (e.g. [42, 43]), and it is striking that Tactopoda *sensu* [3] is never retrieved. The fact that our Bayesian topology (similar to [42]) does not find tardigrades to be close to euarthropods, but that our parsimony-based topology does, could therefore ultimately reflect a methodological conflict. As mentioned above, however, this issue is not surprising given the highly derived condition of tardigrades.

Apart from the Tactopoda versus Onychophora + Arthropod controversy, the main discrepancy between the two topologies lies within the morpho-functional interpretation for the origin and earliest radiation of Panarthropoda. While parsimony favours a secondarily-derived morphological condition based on suspension-feeding for luolishaniids and hallucigeniids with an ancestral “crawling” body plan, a Bayesian approach on the opposite supports the hypothesis that a semi-sessile and suspension-feeding lifestyle might have been the ancestral condition of all panarthropods. Considering growing evidence that a Bayesian approach to discrete morphological matrices generally outperforms parsimony ([44, 45]), as well as the fact that our Bayesian topology is more resilient to changes than our parsimony-based one and a greater overall credibility in the position of onychophorans relative to arthropods, we choose here to present the Bayesian cladogram as our main result and the parsimony as secondary (Additional file 10: Figure S1).

### Disparity analysis

**Fig. 5:**
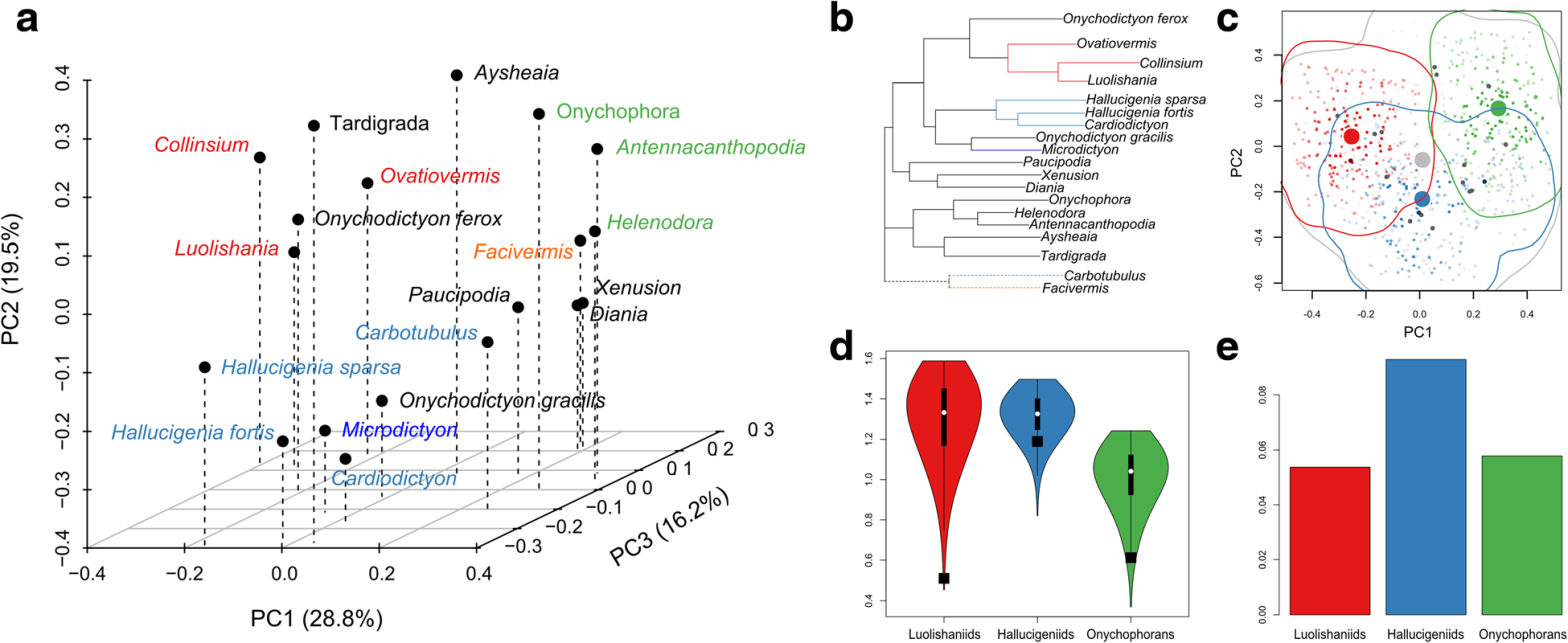
Disparity analysis of total-group Onychophora and lobopodian taxa (19 taxa, 39 characters; see Additional file 10 for a description of the procedure). Colours correspond to clades as retrieved by phylogenetic analyses (Fig. 3). **a** PCoA (morphospace) of the first three dimensions. **b** neighbour-joining analysis of the dissimilarity matrix. Duplicated taxa have been removed and branch lengths averaged. **c** axes 1 and 2 of the hypervolume plot of the first four PCoA. *Coloured clusters* (see (a)) represent uniformly random points sampled from a kernel density estimate of the observed data (*black dots*). Group centroids are represented by *large circles* *. Contour lines* are drawn based on a five percent quantile threshold of projected data using a two-dimensional kernel density estimator (*kde2d*, *R* package *MASS*). * Grey contour line and dots* represent the total data. **d** Violin plots of the jackknifed sums of ranges for the three clades of interest. Observed values are represented by *black squares*. **e** hypervolume as calculated from (**c**) for each clade

We find that onychophoran-like taxa (a total-group Onychophora) (O), luolishaniids (L) and hallucigeniids (H) occupy distinct sectors of the morphospace (Fig. 5a; see also Additional file 10 for a detailed explanation of the procedure)

. The phylogeny-based groups are quasi non-overlapping over the first four dimensions of the PCoA (Sorensen indices > 0.06 in all cases). In addition, based on hypervolume calculations (Fig. 5c), the centroids of all three groups are nearly equidistant (Euclidean distance: L-H = 0.52; H-O = 0.52; L-O = 0.65). A neighbour-joining analysis finds, however, that hallucigeniids cluster with luolishaniids (Fig. 5b). Likewise, a k-means clustering is found to be significant for two groups at four dimensions, one of which is composed of hallucigeniid and luolishaniid taxa.

Hallucigeniids occupy a greater hypervolume of morphospace than both onychophoran-like taxa and luolishaniids (Fig. 5e), while luolishaniids and hallucigeniids both encompass a relatively larger disparity based on sums of ranges than onychophoran-like taxa (Fig. 5d). The sums of ranges between hallucigenids and luolishaniids are not statistically different based on the simulated data (Additional file 10). The hallucigeniid and luolishaniid clusters are therefore more “stretched” along some axes (as is evident, e.g., on axis 1, Fig. 5a, c), while the hallucigeniid cluster is also larger overall (Fig. 5c, e, Additional file 10). Therefore, based on current palaeontological evidence, there is no indication that luolishaniids displayed a significantly greater level of disparity than hallucigeniids, and did not encompass a greater volume of morphospace than total-group Onychophora, providing an appropriate dissimilarity index is used and missing-data-rich outliers are removed (see Additional file 10 for discussion). Notwithstanding, the shape of the hallucigeniid cluster could be somewhat distorted by the less well-known *Carbotubulus*, which lies outside of the hallucigeniid cluster based on neighbour-joining. Moreover, the exclusion of *Diania* and *Xenusion* from the “onychophoran” cluster based on paraphyly is clearly amputating the “bottom-dwelling” early lobopodian body plan from a portion of its achieved morphological diversity.

Owing to the scarcity of the data, it is therefore difficult to conclude that the body plan represented by total-group Onychophora has been drastically less successful (if less spectacular) in occupying morphospace than hallucigeniids and luolishaniids. Certainly, a profound change in ecological niche could have been responsible for the observed differences in disparity: indeed, adaptive opportunities might have been greater for substrate-attached suspension-feeders in Cambrian seas compared to more cryptic bottom-dwellers. Nonetheless, these three groups seem to have similarly explored their own regions of morphospace, with variations involving different parts of their anatomy, although perhaps not over the same time scale. Even if our analysis is somewhat conservative in that a number of autapomorphic features of either Tardigrada or Onychophora (such as the slime papillae or the internalized jaws) were not coded originally for the phylogenetic analysis and thus were not used for the computation of disparity, the addition of these autapomorphies to the matrix does not change our disparity results (see Additional file 10 for discussion). Following graphical observations and the phenogram obtained by neighbour joining (Fig. 5b), inclusion tests also find that *Microdictyon* and *Onychodictyon gracilis* belong to the hallucigeniid morphospace, and that *Onychodictyon ferox* belongs to the luolishaniiid morphospace. Despite displaying more intermediate anatomies, as phylogenies suggest, these taxa have nonetheless clear morphological affinities that do not constitute exploration of non-occupied morphospace between the main groups of interest. This implies that the niches occupied by luolishaniids and hallucigeniids were truly distinct and as such able to drive specific morphological differentiations. The ecological nature of this difference is for now unclear (see below).

## Discussion

Morphological specializations in *O. cribratus* indicate that this animal was most likely well adapted to a sedentary epifaunal, suspension-feeding lifestyle, its habitus being remarkably analogous to that of the Caprellidae, a family of highly specialized amphipods (Crustacea) [46]. The presence of single strong curved claws borne by differentiated, stout lobopods at the back and long spinules at the front evinces that this species, like other luolishaniids [7, 9, 33], was better adapted to anchoring itself on hard substrates than walking on seafloors. Such a feature, strikingly convergent with the three last pereopod pairs of caprellids (whose posterior claws are also stout and turned forward), would have allowed for the anterior portion of the body to be erected in the water column. Outside of the luolishaniids, other lobopodians including *Onychodictyon*, *Hallucigenia* (fig. 2d and extended data fig. 7 in [4]) and *Microdictyon* all share the characters of posterior anterior-facing claws (not only *O. ferox*, as in [9]; see also [39]) and a truncation of the posterior end of the body (*contra* previous interpretations [7, 9, 47] see discussion above and in Additional file 10). This suggests that other lobopodians had evolved some form of posterior differentiation possibly involving the erection of the body. The retention of the anterior-directed claw morphology, but not of single pairs of stout claws in tardigrades was accompanied by miniaturization and other arguably progenetic transformations [38–40] leading to changes in ecology [48], though it is still associated with the grasping of substrates [39].

We construe that such posterior differentiation evolved in concert with the transformation of anterior appendages into collecting and/or filtering apparatuses (Additional files 13 and 14). This is similar to what has been proposed for *Luolishania* [7] and *Collinsium* [9] where all the elongate and spinose lobopods might have formed a sieving device. Such a device was also probably present in the “Collins monster,” another species from the Burgess Shale [14], *Acinocricus* [12] and an unnamed form from Emu Bay [33]. In *O. cribratus,* the six pairs of spinous lobopods would probably have been able to rotate in various orientations, similar to *Luolishania* [7], indicated by the fact that the six pairs are spread out with the first and sixth pairs forming a 90° angle and, like the rest of the body, which shows soft-deformations, were evidently flexible. Assuming such flexibility, the mouth would have been able to reach food particles gathered closer to the body axis by each of the spinulose lobopods, its toothed proboscis functioning similarly to that of priapulid worms [49]. Likewise, caprellids use setae present on their second antenna for suspension feeding [50]; the greater the setation, the greater the effectiveness in suspension feeding [51], although more recent studies show that a wide range of feeding behaviors are possible and that most caprellids, including many with setae, are considered—at least partially—to be detritivores [52]. Accordingly, it is conceivable that *O. cribratus* might have occasionally fed on deposited particles, even though body plan adaptations point to a primarily obligate sessile, suspension-feeding lifestyle.

Compared with other luolishaniids, the few anchoring lobopods in *O. cribratus* and the presence of only two more elongate pairs anteriorly represent both a simplification and further specialization of known morphologies. While anterior and posterior ends of the body in the luolishaniid from Emu Bay Shale remain unknown [33], *Collinsium* [9] is much more elongate, with possibly up to nine non-setose limb pairs forming the anchoring portion of the body—at the very least, appendage pairs 12 to 15, though the morphology of the limbs in the mid-section is less clear. The contrast is even stronger with *Luolishania,* whose limb differentiation is very gradual throughout. Our cladograms suggest that the condition in *O. cribratus* is derived.

Assuming a sieving function of the spinulose appendages, the size of food particles caught— less than 0.2 mm— would be consistent with micro- to meso-nektobenthic prey items in the water column [53] and less likely to be phytoplankton, since the distance between spinules is much wider than the typically small (<75 μm diameter) acanthomorphs found in Cambrian deposits worldwide [54]. In *Collinsium,* also interpreted as a suspension feeder [9], the distance between spinules (~0.15–0.25 mm) and length of the spinules (1.5–2.5 mm) are comparable to the EBS form and *O. cribratus. Luolishania* (Figure 9D in [7]) displays a preserved length of spinules ~0.15 mm, and notwithstanding taphonomic error [55], significantly shorter than that of other luolishaniids, although the distance between spinules of 0.15 mm is comparable with other forms. This shows that different luolishaniids were likely capable of preying on similar types of prey items.

Compared to luolishaniids, hallucigeniids have diversified with a different type of anteriorization, likely not involving sieving, although it is difficult to determine whether their anterior tentacles (or “appendicules” as Ramsköld [47] called them) were involved more in a sensory or food-manipulating function (in, e.g., *Hallucigenia sparsa* [4], they are long enough to reach the mouth). Likewise, their contrasting types of “head” differentiation (elongate or bulbous) could be associated with different ecologies, whether feeding while erected in the water column or grazing. However, in light of our phylogenetic results, a body of morphological evidence suggests that primitive lobopodians, including Hallucigeniidae, were likely epibenthic suspension feeders:

1. Although differing in nature, luolishaniids and hallucigeniids have developed an anteriorization based on limb elongation, coupled with the shared truncated posterior trunk end and the single, anterior-directed posterior claws—seemingly a ground pattern for all lobopodians except *Paucipodia* and *Facivermis*;
2. Part of the lobopodian ground pattern is also the presence throughout the body of elongate lobopods, which is not strictly incompatible with seafloor walking (it could be reminiscent in overall shape and size of the hypertrophied tube feet of some deep sea holothurians that are used for walking [56]), but would be better suited to grasping or anchoring to substrate;
3. Among hallucigeniids, at least *Hallucigenia sparsa* [4] developed claws of a large relative size, not only, as in luolishaniids, on the posteriormost lobopods, but on other trunk lobopods as well; such claws seem ill-suited to elongate, soft limbs, unless they are used for anchoring, climbing or grabbing—a view shared by Steiner et al. [27];
4. Sclerites, as defensive structures, are not indicative of a benthic or epibenthic lifestyle, but their prominent presence in both luolishaniids and hallucigeniids in parallel with their later reduction in “short-legged” lobopodians (with exceptions using different strategies, such as *Diania*) suggests that sclerites and lobopod lengths were possibly correlated (the peculiar case of *Ovatiovermis* is discussed later);
5. Sizewise, hallucigeniids and luolishaniids can be moderately large (*Acinocricus* could reach ca. 10 cm), but they are small enough to live on the surrounding substrates—e.g. sponges; by comparison, "kerygmachelids" are much larger (20 cm or more).
6. Whether the ancestor of panarthropods has more of a nematode or priapulid morphology remains difficult to resolve [57, 58] and the fossil record itself is ambiguous: while *Paucipodia* seems to be little more than a legged nematode, priapulid-like proboscides and palaeoscolecid-like nodes are found in other taxa, suggesting then that the hypothetical panarthropod ancestor combined different cycloneuralian morphologies. As such, the ancestral ecology is also not well constrained. A possible panarthropod transition form could be embodied by the (albeit poorly known) taxon *Facivermis*, from which endobenthic vermiforn panarthropods could have moved to epibenthic ecologies [27]. As Steiner et al. noted [27], the posterior hooks of *Cricoscomia* are reminiscent of the strong claws of certain lobopodians, and in particular, of the stouter single posterior claws observed across the long-legged forms, which hints at their possible origin.
7. Although not preserved in great detail, *Carbotubulus* [29] provides evidence that a typically hallucigeniid morphology—an elongate “head” bearing thin tentacular limbs—could be combined with a luolishaniid trait, namely the thickening of posteriormost lobopods. As such, this taxon strongly reinforces the possibility that both groups shared an epibenthic, suspension-feeding niche.

Given our Bayesian tree, the absence of any scales or spines along the trunk of *Ovatiovermis* could be plesiomorphic, but we construe that dorso-lateral sclerites were more likely present in the common ancestor of luolishaniids and hallucigeniids (as opposed to having sclerites being convergent between these clades). The evolution of biomineralized elements, including skeletonized dorsal elements, in lobopodians is generally viewed as a direct response to the evolution of predators in Cambrian communities, e.g. [9, 59], and, in the case of *Luolishania* [7], and more particularly *Collinsium,* an argument has been made that the presence of spines was a direct consequence of the particular lifestyles of these animals more readily exposing them to predators [9] in presumably more open waters [13]. Several Cambrian lobopodians, notwithstanding, lack dorsal spines or plates, including forms with short lobopods such as *Aysheaia* [17] and *Antennacanthodia* [2]. This begs the question as to how, or if, these “naked” forms, especially the sessile or slow moving ones, adapted to the selective pressure of predation. *Aysheaia pedunculata* was interpreted to have used its claws to potentially cling to sponges, which have also been interpreted as a potential food source, since many specimens are found preserved associated with or close to sponge remains [17]. It was further suggested that, because *Aysheaia* did not bear any spines on its body, it might have also lived in sponge colonies to avoid predators [17]. A similar lifestyle could be inferred for *O. cribratus*, although the paucity of the material precludes the identification of *in situ* associations. Nonetheless, the bed assemblage from which this specimen comes contains numerous sponges, mostly *Hazelia* [37], which could have served as a substrate. As an alternative to physical barriers to predation, camouflage or mimicry have been proposed as potential mechanisms for Burgess Shale molluscs [60] and brachiopods [61]. While protection by sponges, as imagined for *Aysheaia* [17], remains a possibility, and, setting aside a speedy retreat as a defensive action, such a strong morphological reversal could also mean that *O. cribratus* relied on colour-based dissuasiveness (Fig. 2b), a form of aposematism [62], and/or that it was potentially toxic or distasteful to predators. Although difficult to substantiate based on fossil material, and thus less discussed, *Ovatiovermis* further illustrates the fact that the Cambrian response of organisms to the arms race was not exclusively sclerotic or shelly.

## Conclusions

Our study evinces the importance of suspension feeding in the diversification of Cambrian faunas has recently been emphasized, in particular with respect to other panarthropods [9, 53, 63], widening our ecological perspective on fossil forms traditionally viewed as active predators or scavengers. Using a Bayesian approach to lobopodian relationships and revising the identity of the hallucigeniid body plan, we provide here evidence for the plesiomorphic condition of semi-sessile, suspension-feeding lifestyles in all Panarthropoda. This indirectly supports the view that primary producers and mesozooplankton must have been abundant in Cambrian communities. Acritarchs [54, 64] would have been actively preyed or filter-fed upon by the mesozooplankton [65, 66] and small macro-organisms [63, 67]. The mesozooplankton in turn would have been consumed by larger pelagic suspension feeders such as anomalocaridids [53] in the water column, and by other animals, such as luolishaniid and hallucigeniid lobopodians, in the benthos. Thus, not only was the presence of plankton a catalyst of morphological change, but it also precipitated the evolution of a modern style of feeding interaction and food web organization soon after the emergence of animals during the Cambrian period [68].

## Additional files

Additional file 1:
*Ovatiovermis cribratus* from the Burgess Shale, Royal Ontario Museum (ROM) 52707. Part photographed under dry conditions and using direct light (a), or cross-polarized light (b). Scale bars: 5 mm. (JPG 14172 kb)
Additional file 2:
*Ovatiovermis cribratus* from the Burgess Shale, Royal Ontario Museum (ROM) 52707. Part photographed under wet conditions and using direct light (a), or cross-polarized light (b). Scale bars: 5 mm. (JPG 13278 kb)
Additional file 3:
*Ovatiovermis cribratus* from the Burgess Shale, Royal Ontario Museum (ROM) 52707. Counterpart photographed under dry (top) or wet (bottom) conditions and using direct light (a, c), or cross-polarized light (b, d). Scale bars: 5 mm. (JPG 19937 kb)
Additional file 4:
*Ovatiovermis cribratus* from the Burgess Shale, Royal Ontario Museum (ROM) 52707. Close-up of the posterior end of the part photographed under dry (top) and wet conditions (middle and bottom) and using polarized light (a, c), or direct light (b). Scale bars: 5 mm. (JPG 19892 kb)
Additional file 5:
*Ovatiovermis cribratus* from the Burgess Shale, Royal Ontario Museum (ROM) 52707. Close-up of the front end of the part photographed under wet conditions and using direct light (a), or cross-polarized light (b). Scale bars: 5 mm. (JPG 19688 kb)
Additional file 6: Elemental maps of *Ovatiovermis cribratus* (part only) from the Burgess Shale, Royal Ontario Museum (ROM) 52707 before preparation of the 8th left lobopod (lL^8^—see Fig. 1h): carbon (a), calcium (b), silicon (c), potassium (d), aluminum (e), magnesium (f), iron (g), oxygen (h), carbon + calcium (i). The lighter colours represent higher concentrations of elements. C, claw; G, gut; Lu; foregut lumen; M, mouth; Pr, proboscis. Scale bars: 5 mm (a–i). (JPG 16256 kb)
Additional file 7: Scanning electron microscopy images of *Ovatiovermis cribratus* (part) from the Burgess Shale, Royal Ontario Museum (ROM) 52707 in secondary electron mode before preparation of the 8th left lobopod, lL^8^. Scale bar: 5 mm. (JPG 17624 kb)
Additional file 8: Scanning electron microscopy images of *Ovatiovermis cribratus* (counterpart) from the Burgess Shale, Royal Ontario Museum (ROM) 52707 in secondary electron mode. Scale bar: 5 mm. (JPG 16827 kb)
Additional file 9: Paratype of *Ovatiovermis cribratus* from the Burgess Shale, Royal Ontario Museum (ROM) 64006. Scale bar: 2 mm. (JPG 10913 kb)
Additional file 10: Phylogenetic and morphometric analyses, procedural details, datasets, list of characters and additional discussions. (DOC 7074 kb)
Additional file 11: Coding of characters and list of taxa used in this study. (XLS 38 kb)
Additional file 12: Nexus file used for the phylogenetic analysis. (NEX 5 kb)
Additional file 13: Reconstruction of *Ovatiovermis cribratus* showing its purported anchored position and frontal lobopods for suspension feeding. Rotating model. Life reconstruction. (Animation by Lars Fields). (AVI 2836 kb)
Additional file 14: Reconstruction of *Ovatiovermis cribratus* showing its purported anchored position and frontal lobopods for suspension feeding. Life reconstruction. (Animation by Lars Fields). (AVI 3075 kb)
