# Supplementary figures and images for: Cambrian suspension-feeding lobopodians and the early radiation of panarthropods
Source: BMC Evol Biol. 2017 Jan 31;17:29. doi: 10.1186/s12862-016-0858-y (PMC5282736; doi:10.1186/s12862-016-0858-y)

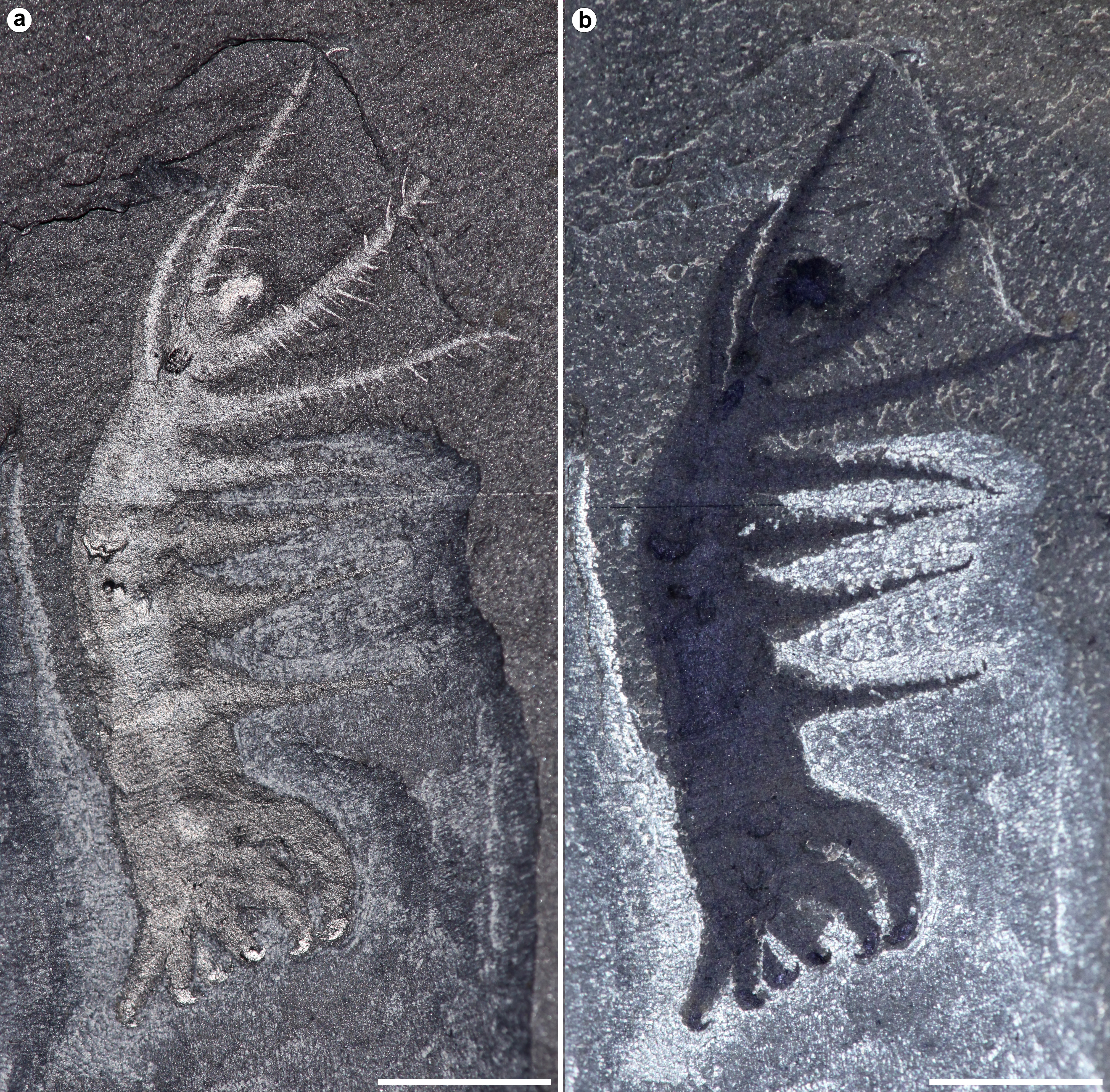

Supplement: Additional file 1: — Ovatiovermis cribratus from the Burgess Shale, Royal Ontario Museum (ROM) 52707. Part photographed under dry conditions and using direct light (a), or cross-polarized light (b). Scale bars: 5 mm. (JPG 14172 kb) [file 12862_2016_858_MOESM1_ESM.jpg]

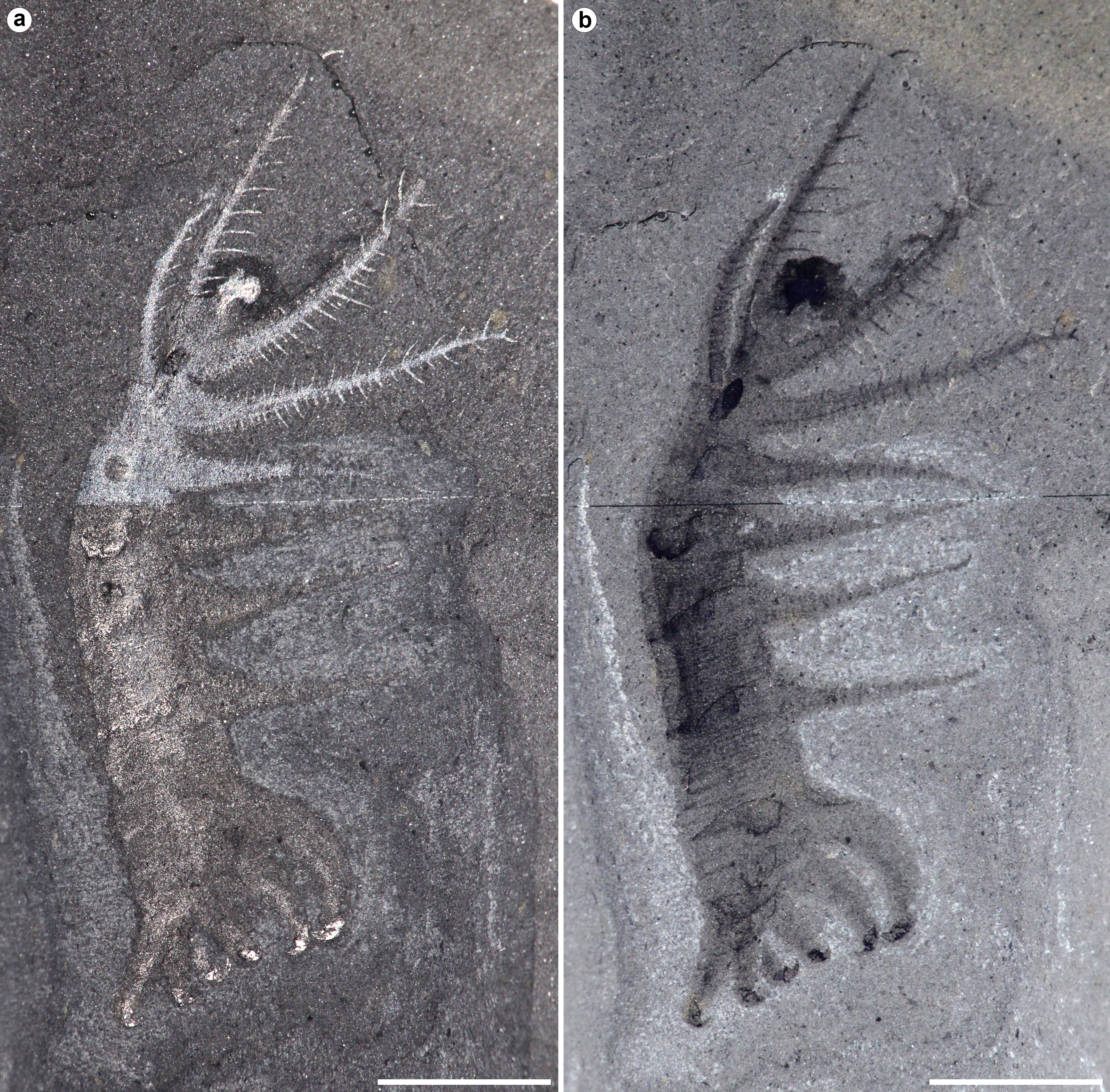

Supplement: Additional file 2: — Ovatiovermis cribratus from the Burgess Shale, Royal Ontario Museum (ROM) 52707. Part photographed under wet conditions and using direct light (a), or cross-polarized light (b). Scale bars: 5 mm. (JPG 13278 kb) [file 12862_2016_858_MOESM2_ESM.jpg]

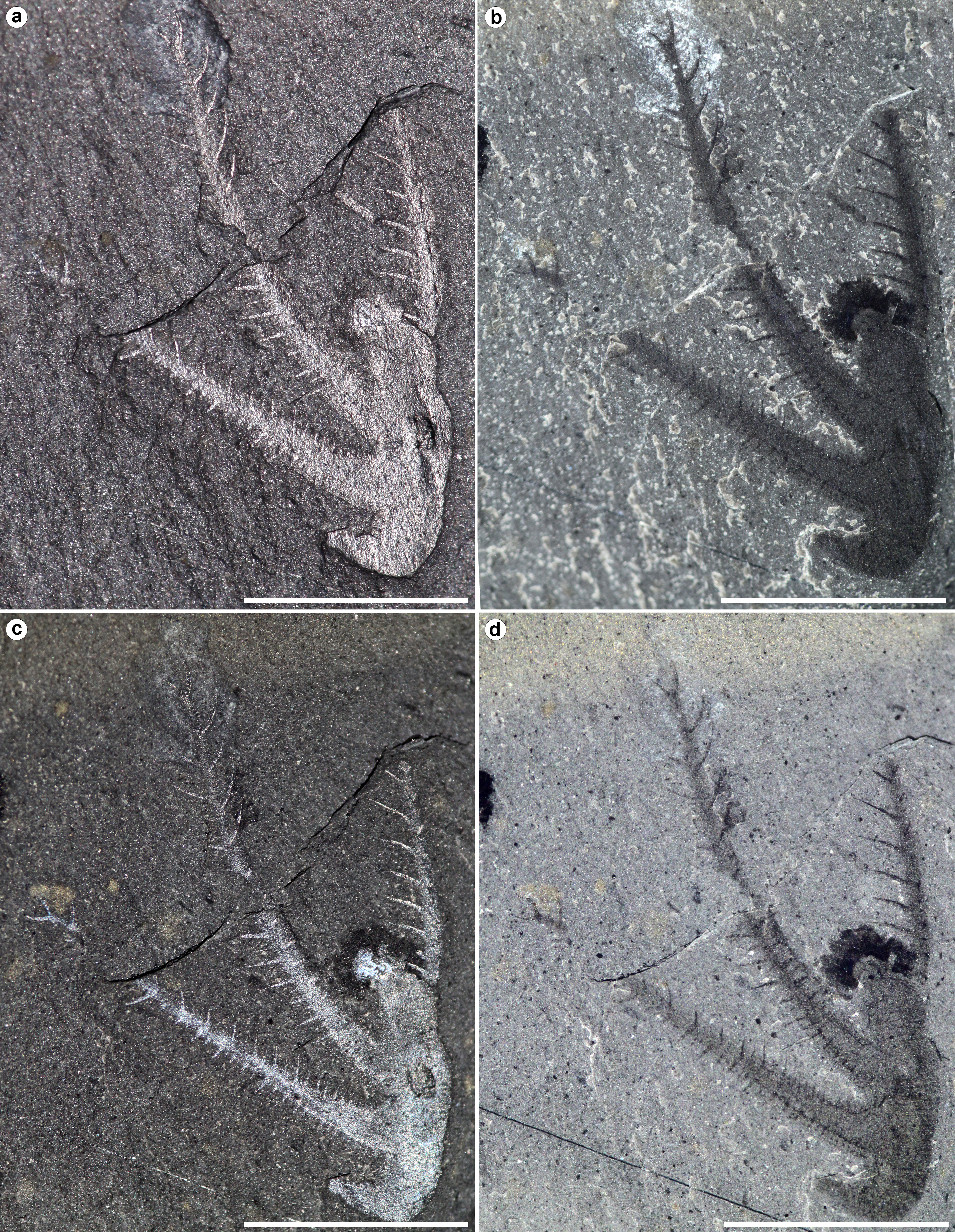

Supplement: Additional file 3: — Ovatiovermis cribratus from the Burgess Shale, Royal Ontario Museum (ROM) 52707. Counterpart photographed under dry (top) or wet (bottom) conditions and using direct light (a, c), or cross-polarized light (b, d). Scale bars: 5 mm. (JPG 19937 kb) [file 12862_2016_858_MOESM3_ESM.jpg]

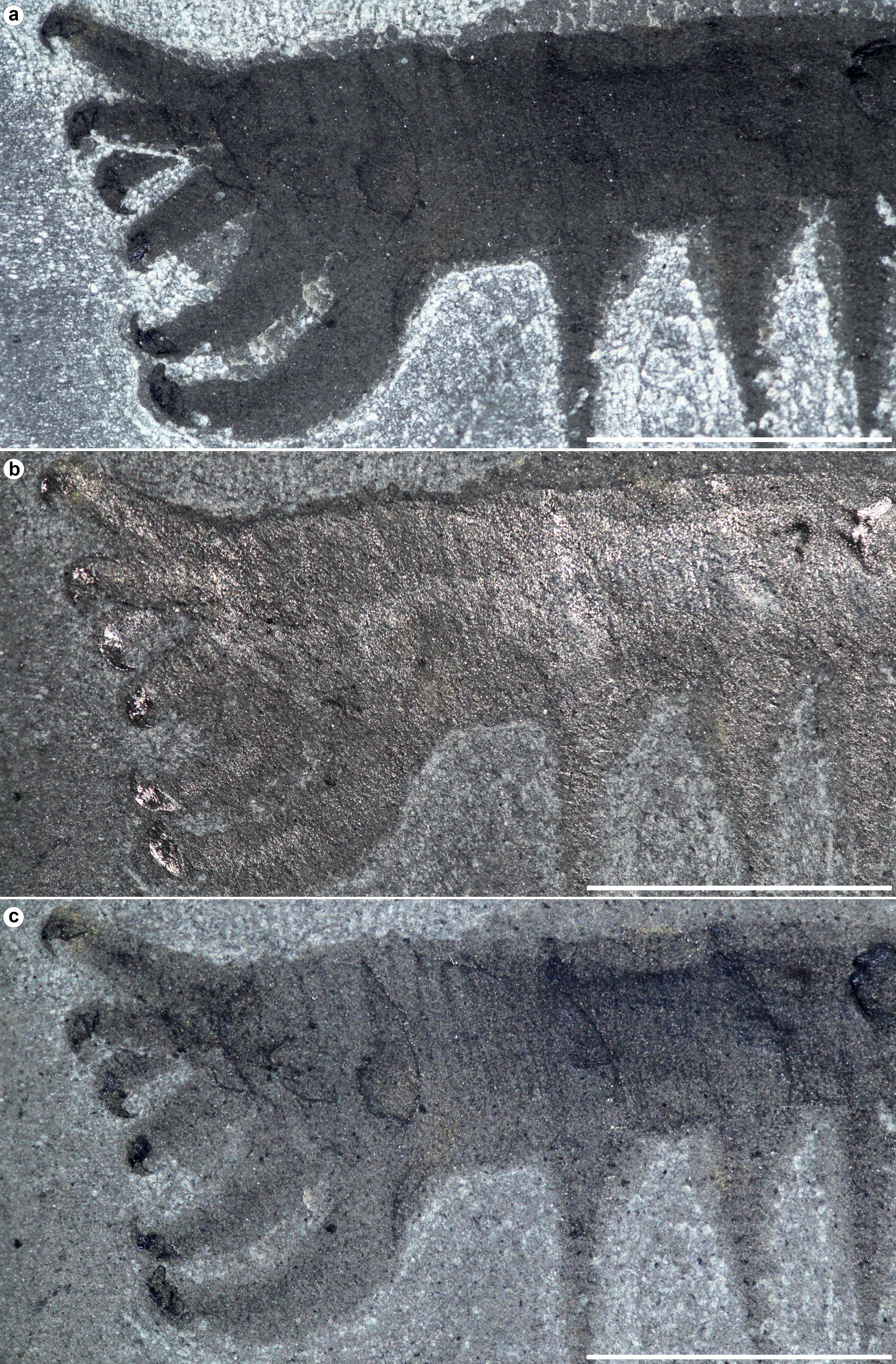

Supplement: Additional file 4: — Ovatiovermis cribratus from the Burgess Shale, Royal Ontario Museum (ROM) 52707. Close-up of the posterior end of the part photographed under dry (top) and wet conditions (middle and bottom) and using polarized light (a, c), or direct light (b). Scale bars: 5 mm. (JPG 19892 kb) [file 12862_2016_858_MOESM4_ESM.jpg]

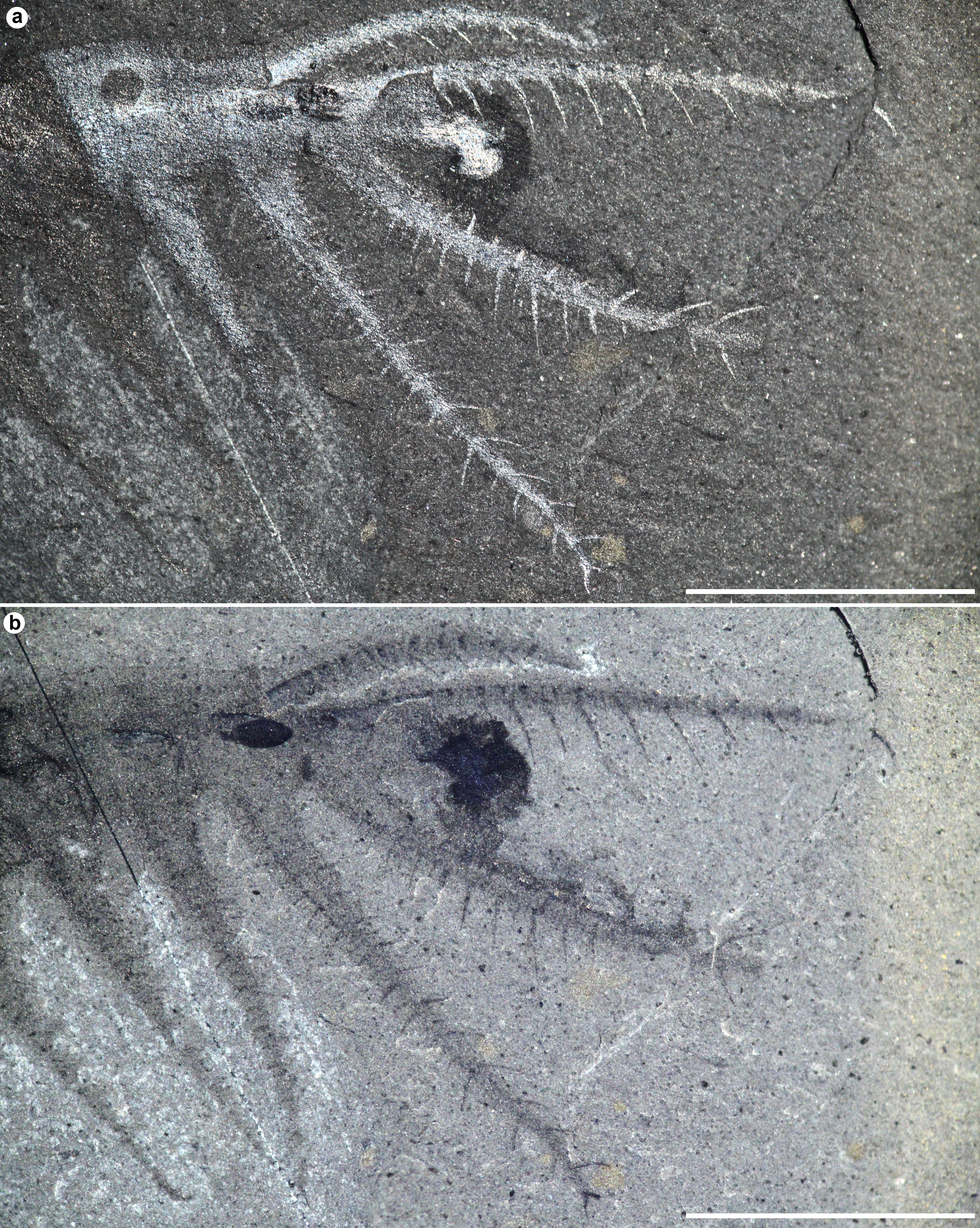

Supplement: Additional file 5: — Ovatiovermis cribratus from the Burgess Shale, Royal Ontario Museum (ROM) 52707. Close-up of the front end of the part photographed under wet conditions and using direct light (a), or cross-polarized light (b). Scale bars: 5 mm. (JPG 19688 kb) [file 12862_2016_858_MOESM5_ESM.jpg]

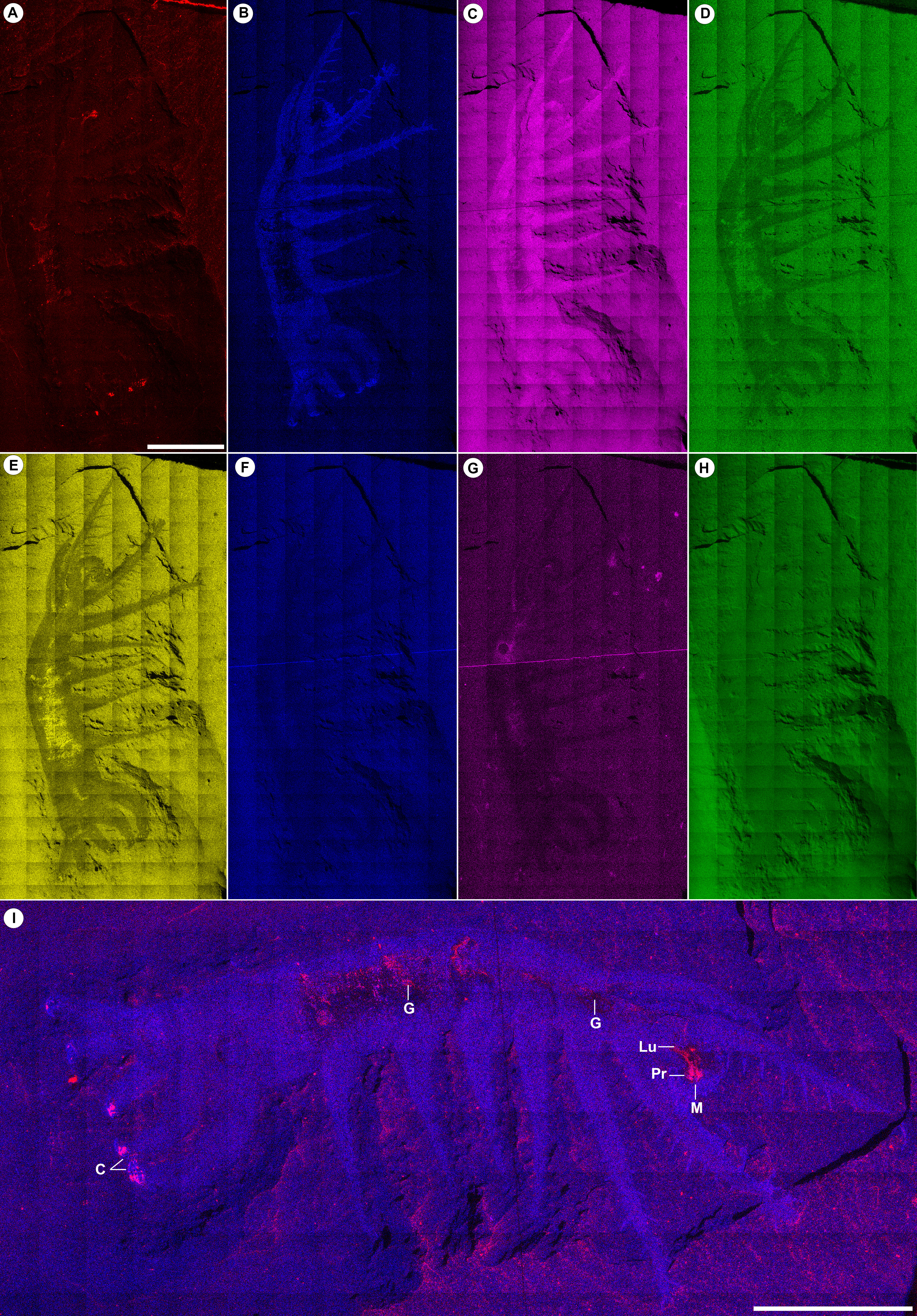

Supplement: Additional file 6: — Elemental maps of Ovatiovermis cribratus (part only) from the Burgess Shale, Royal Ontario Museum (ROM) 52707 before preparation of the 8th left lobopod (lL8—see Fig. 1h): carbon (a), calcium (b), silicon (c), potassium (d), aluminum (e), magnesium (f), iron (g), oxygen (h), carbon + calcium (i). The lighter colours represent higher concentrations of elements. C, claw; G, gut; Lu; foregut lumen; M, mouth; Pr, proboscis. Scale bars: 5 mm (a–i). (JPG 16256 kb) [file 12862_2016_858_MOESM6_ESM.jpg]

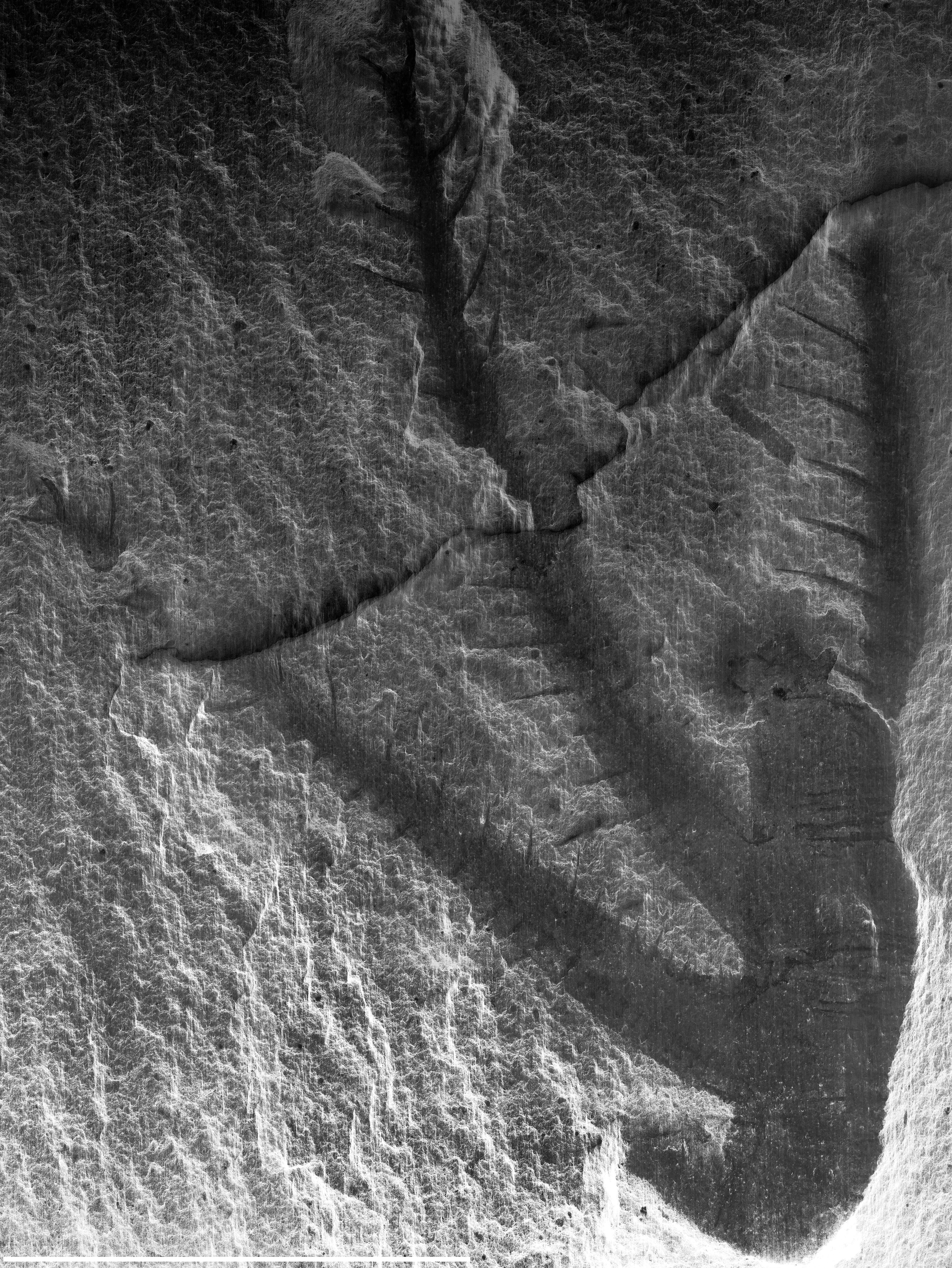

Supplement: Additional file 8: — Scanning electron microscopy images of Ovatiovermis cribratus (counterpart) from the Burgess Shale, Royal Ontario Museum (ROM) 52707 in secondary electron mode. Scale bar: 5 mm. (JPG 16827 kb) [file 12862_2016_858_MOESM8_ESM.jpg]

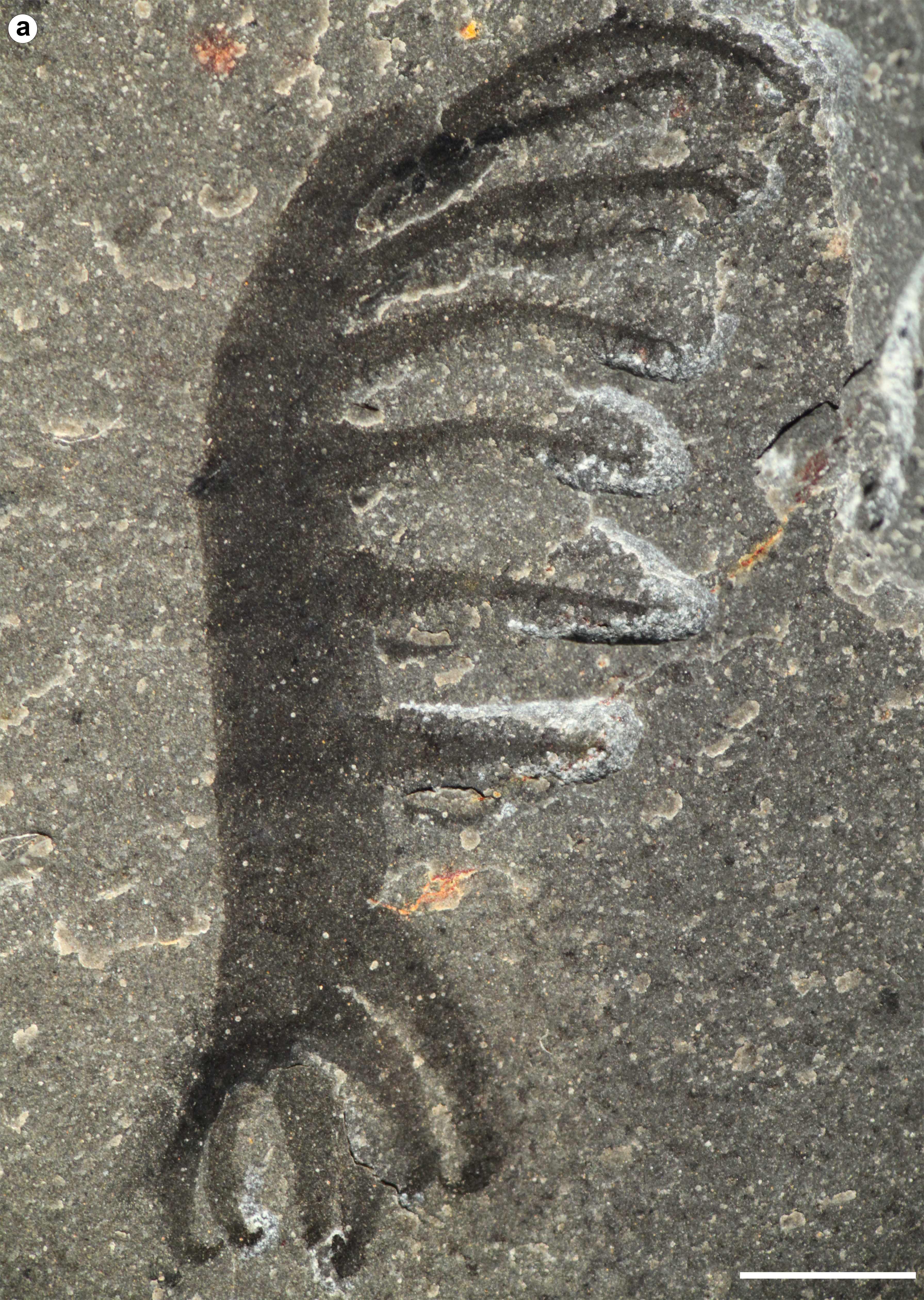

Supplement: Additional file 9: — Paratype of Ovatiovermis cribratus from the Burgess Shale, Royal Ontario Museum (ROM) 64006. Scale bar: 2 mm. (JPG 10913 kb) [file 12862_2016_858_MOESM9_ESM.jpg]
