# Supplementary material for: Cambrian suspension-feeding lobopodians and the early radiation of panarthropods
Source: BMC Evol Biol. 2017 Jan 31;17:29. doi: 10.1186/s12862-016-0858-y (PMC5282736; doi:10.1186/s12862-016-0858-y)
Supplement: Additional file 10: — Phylogenetic and morphometric analyses, procedural details, datasets, list of characters and additional discussions. (DOC 7074 kb) [file 12862_2016_858_MOESM10_ESM.doc]

**Additional File 10**

**Phylogenetic analysIs**

**Phylogenetic tree reconstruction:** All analyses were performed using 38 taxa and 59 characters (**Additional File 11,** see also the nexus file **– Additional File 12**) using MrBayes 3.2.6 for the Bayesian analysis and PAUP*4.0a150 for the parsimony analysis. The Bayesian tree reconstruction followed a Mkv model of likelihood (i.e., the assumption that all observed characters are variable with equal transition rates between states) and used gamma rates (i.e., allowing for variable rates of state change) during two parallel runs of 10,000,000 generations (four chains) with a tree sampled every 1000 generation and burn-in of 20%. The tree search based on parsimony used heuristic search with tree bisection reconnection (TBR), for which we constrained a limit of 10 trees of scores ≥ 1 for each of the 1000 replicates. Characters were unweighted and unordered, and inapplicable entries were treated as missing data. Although this method decreases the stability and resolution of the tree, it avoids overemphasizing the absence of homologous structures .

Major apomorphies for the clades of interest based on the Bayesian topology are as follows:

- Hallucigeniids are unified across all topologies by the possession of slender, tentacular post-oral limbs (c. 10). A tentative apomorphy is the presence of a differentiated head portion (c. 8), which would support the inclusion of *Microdictyon* to this clade.
- A clear apomorphy for luolishaniids is the presence of paired chevron spinules (c. 48). The recruitment of several limbs for anterior differentiation (c. 5) is also well established as an apomorphy of the group, which also applies to *Facivermis*, albeit in an autapomorphic fashion. The presence of a median row of spines (c. 40) appears to be an apomorphy of a restricted luolishaniid clade without *Ovatiovermis*.
- The appearance of “cone-shaped” lobopods (in the “short-legged taxa,” c. 32) is a strong apomorphy of Arthropoda+Onychophora+Tardigrada, including *Kerygmachela*-like taxa.. The loss of the single posterior claws (c. 58) is also a strong candidate as an apomorphy of this clade.
- Changes in mouth orientation, types and structures are also optimized as critical in the transition between the luolishaniid-hallucigeniid stem and more derived panarthropods, although many uncertainties and the status of certain *Kerygmachela*-like taxa do not allow yet for a clear pattern of state changes.
- The development of a larger frontalmost appendage is characteristic of the transition between *Hadranax*/*Xenusion* and Arthropoda+“kerygmacheliids,” as is the appearance of secondary digestive organs.

**Character list and notes on character coding:** The following list of characters and corresponding data set were started in 2010 and expanded in 2012 as part of a larger phylogenetic project on panarthropods [CA]. We have carefully considered other data sets focusing on lobopodian relationships, in particular those of Ramsköld and Chen 1998 , Liu et al. 2011 , Ma *et al*. , Smith and Ortega-Hernández (as well as its modifications ).

Our character coding substantially differs from previous studies, including the latest version of the Smith and Ortega-Hernández data set in Yang *et al*. , because of both interpretative and methodological reasons. We found some characters to be phylogenetically non-informative, either autapomorphic (characters 40, 45 and 48 in ), redundant (character 12 internally; character 68 with 71 in ), constant (character 37 in ), or uncertain for most included taxa (especially characters 81–86 in ). Characters which were the subject of recent publications, namely, the stacked development of sclerites and the paired ganglia constituting the ventral nerve cord , are also difficult to polarize with confidence, as their state remain unknown in most fossil taxa. Because both, however, havebeen proposed as representing important synapomorphies for hallucigeniid-onychophoran and Tactopoda clades respectively, and because we are able to assess the presence of stacked sclerite elements in *Ovatiovermis*’ claws, we nonetheless included these characters in our own analysis. The ganglionic nerve cord in particular was optimized as the only synapomorphy of Tactopoda in Yang *et al*. (their character 59, “secondary structures on lobopodous limbs,” is also optimized as an original synapomorphy of the group, but we do not think the coding is sound—see our comment on characters 47 and 48 below).

In places, we find the reading of some of such evidence by Yang *et al*. to be insufficiently critical. For example, the coding of “number of neuromeres integrated into the dorsal condensed brain” and “mouth innervation relative to brain neuromeres” in *Lyrarapax* seems to go beyond the available palaeontological information. The authors also homologize the second tentacle pair of hallucigeniids with the jaws of onychophorans as deutocerebral, but to our knowledge, there is no evidence to support such a claim, but rather an assumption of direct topological correspondence that we would rather not follow at this point. More problematic to us is the fact that, in assuming such homology, the authors have discarded one of the most important synapomorphies of hallucigeniids, namely, the anterior tentacle-like limbs. Other deviations from this dataset and others are detailed below.

Since our aim was not to dwell on internal relationships within either anomalocaridids—not including, for instance, *Aegirocassis*— characters 7, 13, 40 and 65 in Yang *et al*. were not included. We also limited the incorporation of characters related to neural and circulatory anatomy to avoid adding large blocks of question marks in a data set in which the morphological signal is already scarce. Although those characters, for the most part, could influence the grouping of tardigrades or onychophorans with arthropods, we found more interesting to test the effect of fossils on that issue. To us, this is preferable to exposing our small dataset to the well-documented artifacts of missing data especially as we are treating inapplicable states as uncertainties. In the case of extant onychophorans, we chose to represent them under a single Onychophora taxon in our matrix, as with the current character set the various genera would have had identical coding and would have represented a single point in the disparity analysis. The impact of onychophoran autapomorphies (slime papillae, “jaws”) on the morphospace has also been tested (see below).

We have also modified some characters published by Murdock *et al*. . Murdock *et al*. documented new specimens of *Helenodora*, showing the presence of onychophoran-like antennae and a rounded posterior trunk termination. We disagree with the authors, however, on their interpretation of the absence of jaws and claws based on expectations from decay experiments. Fossils are not simply replicates of decay phases, but the results of many taphonomic factors, including the angle of burial, compaction of superimposed tissues and, as Murdock and colleagues acknowledge, properties specific to the environment of entombment. Claws are an inconsistently preserved panarthropod feature in two-dimensional Lagerstätte, although they are present in all well-documented lobopodians. Despite their rather large relative size, they were initially not accounted for in *Hallucigenia* for instance . The lack of preservation of the claws in *Helenodora* is likely the result of variations in burial angle and/or poor preservation potential for distalmost features. As for jaws, only exceptional conditions would allow for the outline of a small internal cuticular structure to appear clearly at the splitting plane of the part and counterpart of a fossil, which is arguably difficult to obtain if Mazon Creek animals are preserved “as an external mould of the surface of the outer cuticle” , and especially difficult to ascertain in only two specimens preserving a complete head. We therefore maintained coding of uncertainty (“?”) for these characters.

**Comparison of character coding with other analyses:**

When applicable, we provide information pertaining to original discussions of characters, as well as references for alternative coding in case of disagreement. Publication code is as follows: [R1x] Ramsköld 1992 and “x”, order of characters in that publication; [R2x] Ramsköld and Chen 1998 , [Dx] Daley *et al*. 2009 , [M1x] Ma *et al*. 2009 , [M2x] Ma *et al*. 2013 , [Yx]: Yang *et al*. 2015 dataset (note: this dataset is an updated version of a previously published dataset by Smith and Ortega-Hernández 2014 ; this dataset was also modified separately by Smith and Caron 2015 and Murdock *et al*. 2016 ). Additional sources are added as required.

**GENERAL**

1] Paired limbs

1. Absent
2. Present

*Remark*: We use this character as the main ingroup synapomorphy of Panarthropoda (see e.g. M1#1).

2] External subdivision of integument

1. Annulated
2. Segmented

*Remark*: We have here abandoned the concepts of homonomous versus heteronomous (e.g. ) in favour of describing the area of limb insertion in lobopodians (see char. 25). Although the preservation of annuli is inconsistent, frequently varying even within single specimens (e.g., , fig. 14), and may be size dependent (as in *Hallucigenia*, unpublished observations), there is ample evidence (e.g. ) that all lobopodians and onychophorans had more or less sclerotized integumental annulation with “nodes” (char. 35 below), possibly inherited from palaeoscolecid-like ancestors (See M2#30 and Y#36 for equivalents). Note that we are using the term “annuli” in the sense of Ramsköld , not in the sense of Scholtz (See D#25, M2#2)—however, Ramsköld suggested that larger, smooth body sections, could represent a single “annulus.” In onychophorans, annuli are in fact called plicae (e.g. ), and we use this term in conjunction with annuli. We code tardigrades with a “?” as the status of their wrinkled cuticle evades the two states used here.

3] Type of integumental segmentation

1. Segmented but not sclerotic
2. Arthrodized (=tergo-sclerotic)

*Remark*: For taphonomic reasons related to size, the integumental subdivision of anomalocaridid bodies remains unclear beyond the fact that arthrodization is absent (see D#25). Based on *Opabinia* , however, it seems that external segmentation is present, replacing annulation in some basal arthropods, including *Schinderhannes* and isoxyids (e.g. ), but that these segments are not articulating sclerites. Arthrodization throughout refers to the presence of sclerites articulating via arthrodial membranes.

**ANTERIOR SECTION**

4] Anteriorization including differentiated limbs

0. Absent

1. Present

*Remark*: We tentatively homologize the differentiation of anterior limbs as an evolutionary event with a common origin, despite strong differences between types of limb anteriorization in lobopodians (see characters 5, 10 and 11). We take here into account any type of appendage—hence tardigrades are also coded “1” for their stylet.

5] Anteriorization of multiple lobopods

1. Absent
2. Present

*Remark*: Luolishaniids have evolved the differentiation of more than one pair of anterior axial limbs, which contrasts with the primary involvement of a single anteriormost pair in *Aysheaia*, *Onychodictyon*, and large forms such as *Megadictyon* and dinocaridids. Hallucigeniids are also characterized by several anterior pairs of differentiated limbs (we call them “tentacles” after the terminology in ), but we consider that their homology with the anterior lobopods of other taxa remains uncertain, and code their condition as a separate character (10).

6] Somital head (as tagma) defined by strong differentiation of several axial appendage pairs or external cephalization

1. Absent
2. Present

*Remark*: Lobopodians with one or several prominent pair(s) of anterior appendages lack a head tagma per se, in contrast to e.g. *Surusicaris* , in which post-frontal head limbs are strongly differentiated from trunk limbs. The morphology of *Luolishania* and *Collinsium* suggests that the anterior to posterior differentiation is more gradual in luolishaniids, in spite of the well-defined anterior contrast seen in *Ovatiovermis*. The presence of a head tagma in dinocaridids is uncertain .

7] Bases of frontalmost pair of appendages occupying entire “head” portion laterally and adjoining first trunk lobopod pair

1. Absent
2. Present

*Remark*: We consider here the strict “head” of lobopodians as the anteriormost section of body with or without “ocular appendages” (antennae, tentacles) preceding the first pair of trunk lobopods. In *Kerygmachela* , *Pambdelurion* and *Siberion* , the lateral margins of the “head” are fully occupied by the insertion of the frontalmost appendages, and there is no lateral interspace between the anteriormost portion of the body and the trunk. We code *Megadictyon* as uncertain as it is not clear whether such interspace exists in this taxon, whereas *Jianshanopodia* is coded as “0”.

8] Strong modification of head shape in lobopodians, excluding proboscides and appendages

0. Absent

1. Present

*Remark*: Hallucigeniids and *Microdictyon* display very unusual differentiations of the head in either very elongate “necks” or very large, ovoid protrusions (see character 9 below). We exclude here conditions such as the bulbous proboscis of *Onychodictyon ferox*.

9] Strong head differentiation in lobopodians, excluding proboscides, type

1. Single large, ovoid mass much wider than trunk
2. Elongate anterior extension of body bearing head features most distally

*Remark*: There are two distinct deviations from the simplest “head” form in lobopodians: one is a slender elongation, as exemplified by *Microdictyon* and *Hallucigenia sparsa*, and the other is a bulbous thickening of the body, as in *Hallucigenia fortis* and *Cardiodictyon*. The elongate head type is distinct in that the small terminal (anteriormost) portion bears the mouth, pharynx and eyes at a distance from the first pair of trunk lobopods (notwithstanding the occasional tentacles) that is at least three times as great as the interval between lobopod pairs 1 and 2. By contrast, the “ovoid” head type is characterized by a great swelling of the anterior portion forming a large bulbous structure about twice as wide as the trunk or larger. The distinct identity of elongate hallucigeniid heads was stressed notably by Ramsköld —see also R2#1—but we do not regard *Paucipodia* as presenting this condition. Compared to lobopodians, onychophorans and tardigrades, the head of euarthropods is a more integrated anterior section of the anterior body (a head tagma) defined by the number of differentiated appendage pairs (see char. 14). In spite of the evidence for an early origin of the typical arthropod brain , arthropod taxa are here coded as inapplicable as we make no assumption about the common origin of the external “head” in both groups (see also char. 8).

10] Tentacles

1. Absent
2. Present

*Remark*: *Cardiodictyon* and *Hallucigenia* share the presence of two pairs of ventral tentacle-like appendages distinct in morphology from the trunk limbs. Emphasis was put on those differentiated tentacle-like limbs by Ramsköld and Chen (, char. 14), although we cannot verify their assertion that *Microdictyon* also had elongate anterior limbs. See M2#9 for equivalent. In *Hallucigenia sparsa* , the first trunk limb (corresponding to the first pair of dorso-lateral spines) is modified into an identical tentacle-like limb, which is an autapomorphic condition of that species.

11] Ocular appendage

1. Present
2. Absent

*Remark*: Evidence has recently been presented to support the protocerebral affinity of the dinocaridid frontal appendage ( but see ). This, in conjunction with the protocerebral identity of the onychophoran antenna , would favour a protocerebral interpretation of all similar antennular frontal appendages in lobopodians, in contrast to euarthropods in which the deutocerebral identity of the frontalmost appendage is now widely accepted . Given evidence of the appendicular origin of the labrum (e.g. ), some have hypothesized that this structure is a vestigial protocerebral appendage of the type found in early panarthropods. There are still issues with this scenario, however, such as the fact that morphological intermediates between anomalocaridids and megacheirans do not show evidence for a transition of main frontal appendage type, while already displaying a ground-pattern head tagma . Putative transitional forms such as large bivalved arthropods are not sufficiently known to be confident about their basal phylogenetic placement . As a result, the moment of such transition from proto- to deutocerebral main frontal appendage remains uncertain, and it is for now safer to rely on external, topological information rather than assumed neurological one. This character thus codes for the presence of an antenna-like frontal-most appendage in lobopodians, that is, an anteriormost appendage distinct morphologically from trunk lobopods and located near the other main head features – mouth and eyes. We code presence in *Surusicaris* based on general topological continuity and the presence of dinocaridid features on its frontal appendage, although, as mentioned, this conflicts with the ground-pattern type of head in this taxon suggesting rather a deutocerebral affinity of the frontalmost appendage. Accordingly, euarthropods are coded as uncertain.

12] Ocular appendage bearing cuticular projections

0. Absent

1. Present

*Remark*: This character describes tubercle- or seta-like cuticular outgrowths on the antennular lobopodian appendages, such as the “branches” of *Aysheaia*’s frontal appendage .

13] Projections type on ocular appendage

1. Soft
2. Sclerotized, spines

*Remark*: As in *Aysheaia*, projections on large *Kerygmachela*-like lobopodians appear much softer and more tubercular than the inner spines of dinocaridid and *Surusicaris* frontalmost appendages.

14] Hypertrophied, raptorial ocular appendage

0. Absent

1. Present

*Remark*: *Kerygmachela*-like lobopodians and dinocaridids have an ocular appendage that is significantly enlarged relatively to other limbs, with a commonly raptorial function, although other specializations of this limb had evolved . As per our comment in char. 11 and main text, we leave the “great appendage” of megacheirans as uncertain.

[15] Arthrodization of any appendages

0. Absent

1. Present

*Remark*: We code here a likely developmental homology, in spite of proposed topological differences between dinocaridid and euarthropod frontalmost appendages (see also char. 11 and main text).

16] Arthrodized frontalmost appendage antennular

0. Absent

1. Present

*Remark*: This character differentiates the antennule in *Olenoides* and *Chengjiangocaris* from the “great appendage” of *Yawunik*. A more detailed reappraisal of the phylogenetic relationships between these taxa is in preparation by the authors.

17] Sclerites on anteriormost section of body axis in lobopodians

0. Absent

1. Present

*Remark*: Although this has been contested , *Cardiodictyon catenulum* and *Hallucigenia fortis* have been described with of a pair of sclerotic plates covering the anterior, bulging part of the body . Their well-defined ovoid shape is recurrent across specimens of both species, and, in several cases, tend to preserve in the same aspect as sclerites (e.g. Fig. 14.5a in ; Fig. 2B in ); we therefore maintain the original interpretation (as in M2#37). Smaller paired sclerites have been described in *Luolishania* and *Collinsium* (); their difference in size from the head plates in *Cardiodictyon* and *Hallucigenia* *fortis* is coded in the following character [18]. Given the absence of evidence for a homology between these paired sclerites and the tergal elements of arthropods, we code them as separate characters.

18] Size of head sclerites in lobopodians

0. Large (covering most of the area of the “head”)

1. Small (covering about half or less of the “head”)

19 Tergal sclerotization of the head

1. Absent
2. Present

*Remark*: This character accounts for the presence of any cephalic tergite, including those forming carapaces and shields, and is only coded as present in arthropods. A tergite is understood as a single dorsal cuticular plate and is therefore not homologized with the paired lateral sclerites found in some lobopodians (character 17).

20] Inter-ocular tergite

1. Absent
2. Present

*Remark*: This character specifically codes for the presence of a single, dorsal, inter-ocular tergite—usually referred to in stem arthropods as the “anterior sclerite” (e.g. ). This is similar to char. 4 in .

21] Visual organs

0. Absent

1. Present

*Remark*: Ma *et al*. argued for an evolutionary continuity between lobopodian and arthropod visual organs. This character codes for such a hypothesis.

22] Visual organs, type

1. Ocelli
2. Compound

*Remark*: We have grouped all ocular features known in lobopodians under the single denomination of ocelli due to lack of documentation of the specific types described by Ma *et al*. in many taxa.

**MOUTH AND DIGESTIVE TRACT**

23] Mouth opening anteriorly

1. Absent
2. Present

*Remark*: The ventral mouth in the onychophoran sense is a mouth opening fully located on the ventral surface of the main body axis, at the level of limb insertion. Contrarily to Yang *et al*. (their char. 23), we do not recognize evidence of a ventral mouth among “long-legged” lobopodian taxa for which this character can be assessed. The most recent redescription of *Hallucigenia sparsa* , in fact, reconstructs the mouth as anterior, not ventral. The further differentiation between ventral and posterior transitions is not clear among arthropods. See M2#21 for equivalent.

24. Circumoral structures

0. Absent

1. Present

*Remark*: The evolution of this character in Ecdysozoa was recently discussed by Smith and Caron . In contrast to cycloneuralians and basal panarthropods, the condition in euarthropods is arguably highly modified as mouth sclerites are not circumoral but pre-oral, composed, when known, of the labrum-hypostome complex whose debated origin is not tackled here.

25] Type of circumoral structures

1. Toothed lips
2. Lamellae
3. Plates

*Remark*: This character has been the focus of a recent analysis . Although it is not clear whether the small oblong juxtaposed sclerotic elements in *Hallucigenia sparsa* form a full circumoral ring, we follow the authors and homologize them with those of dinocaridids. We emphasize, however, with the following character (24), that these elements are internal rather than external (i..e “circumoesophagal” as opposed to circumoral). Although “peytoia-like mouthparts” have been described from *Megadictyon* , we doubt their authenticity based on published evidence . The circumoral lips of onychophorans are morphologically akin to those of tardigrades, in which they can be more or less plate-like . We consider that the condition is homologous in *Aysheaia*. Although the onychophoran circumoral papillae are likely autapomorphic, as secondarily formed around the deutocerebral appendage and involving innervation from all brain ganglia , at least some of these papillae are still connected to the protocerebrum, as is the case in tardigrades and likely represent the ancestral condition. We thus depart from the approach taken by Yang *et al*. (, Y#25) in regarding circumoral papillae in both taxa as analogous only.

26] Location of circumoral structures

0. Internal (within buccal cavity)

1. External (outside buccal cavity)

*Remark*: In *Hallucigenia sparsa*, the circum-oral plates have been shown to be located within the buccal cavity .

27] Proboscis

1. Absent
2. Present

*Remark*: We take here into account the criticism in Ma *et al*. of the original interpretation of *Diania* and coded a proboscis as uncertain. We likewise coded uncertainty for a proboscis in *Aysheaia* based on controversy about the presence of this structure . As seems to be the case in *Collinsium*, *Ovatiovermis* possesses a proboscis-like soft structure anterior to the pharynx equipped with numerous rows of teeth, somewhat similar to the priapulid proboscis.

28] Secondary organs connected to the central digestive duct

1. Absent
2. Present

*Remark*: Similar to Ma *et al*. (char. 42); see also Vannier *et al*. for this character and character 27. Only large lobopodians (e.g. *Jianshanpodia*, *Kerygmachela*) are known to possess secondary digestive structures, although the condition in large luolishaniid taxa remains unclear.

29] Shape of post-cephalic secondary digestive structures

1. Reniform
2. Bulgy triangles
3. Caeca

30] Metameric ganglia on nerve cord

1. Absent
2. Present

*Remark*: Strong homology has been established between the nerve cord of tardigrades and that of euarthropods and potentially constitutes a synapomorphy of Tactopoda .

**TRUNK**

31] Lobopodous limbs (anywhere)

0. Absent

1. Present

*Remark*: We code here exclusively for unsegmented limbs, excluding dinocaridid flaps (char. 25 below). See e.g. M2#2. In conjunction with character 1, we make it implicit here that lobopodous limbs are modified in dinocaridids and euarthropods. We code them as present in *Surusicaris* , although the typical lobopods with annuli are only present in the head. The presence of lobopods in addition to flaps in *Kerygmachela*, *Pambdelurion* and especially *Opabinia* has caused controversy , and it is a difficult issue of overlapping structures . We code lobopods as uncertain in these taxa.

32] Type of main lobopodous trunk limb

1. Short, conical, subequal or shorter than trunk width (“short-legged”)
2. Elongated, significantly longer than trunk width (“long-legged”)

*Remark*: The existence of long-legged morphotypes, as opposed to short-legged morphotypes, has been recognized before among lobopodians, e.g. . We concur with this distinction, regardless of limb differentiation along the body of a single individual. A morphometric analysis of lobopod shape across genera could help redefine this *a priori* important character.

33] Lateral limbs as broad flaps

1. Absent
2. Present

*Remark*: This character is coded as characterizing the dinocaridid body plan, as well as *Kerygmachela* and *Pambdelurion*. As D#36.

34] Arthrodization of trunk limbs

0. Absent

1. Present

*Remark*: This is a synapomorphy of Euarthropoda.

35] Nodes/tubercles/dermal papillae

0. Absent

1. Present

*Remark*: We consider that the tubercles on the scleritome of palaeoscolecids and on the integument of onychophorans and a number of lobopodians are homologous. We code them as absent in *Paucipodia* and hallucigeniids, as there is not, to our knowledge, clear evidence of nodes or tubercles on the annuli/plicae of these taxa. At the very least, these structures are sufficiently reduced or modified to justify the coding of a different state. *Contra* Yang *et al*. (, Y#39), we code spines, plates and other sclerotic structures above limb insertion as a separate character. See also M2#10 and M2#41.

36] Integumental differentiation at limb insertion

1. Absent
2. Present

*Remark*: This character is applicable only to lobopodians because it is incompatible with segmentation. It designates in these taxa any differentiation of the integument at the location of limb insertions (and the corresponding body ring, but not necessarily the entire metamere), whether an interruption in the annulation pattern (as is the case in *Ovatiovermis*) or the presence of cuticular outgrowths. Although there is evidence for differentiation at limb insertion in the form of anastomosing plicae in certain onychophorans , the interruption of the annulation pattern is only partial and we thus code 0 for all onychophorans.

37] Dorso-lateral sclerites above limb insertion

1. Absent
2. Present
3. Large nodes

*Remark*: This character and all following dependencies are applicable only to lobopodians. It could be hypothesized that lobopodian sclerites are developmentally and evolutionarily related to arthropod sclerites . We opted here for a neutral coding and differentiated this character from the possession of tergites/sternites proper. By contrast to other luolishaniids, *Ovatiovermis* lacks sclerites in its entirety.

38] Dorso-lateral sclerites, type

1. Spine
2. Plate

*Remark*: The intermediary condition of “spinose plate” in *Onychodictyon ferox* is coded as polymorphic. We consider the dorsal structures in heterotardigrades to be akin to spinose plates (coded as plates only in Y#41).

39] Length of dorso-lateral spines

1. Short (length inferior to slightly superior to body diameter)
2. Long (at least twice as long as body diameter)

*Remark*: There is a relatively clear dichotomy between the elongate spines of *Hallucigenia* species and the spines of *Onychodictyon ferox*. *Luolishania* is coded as polymorphic.

40] Median spine above limb insertions

1. Absent
2. Present

*Remark*: Armoured luolishaniids (*Luolishania*, *Acinocricus* [not included in this analysis], Emu Bay Shale Collins’ monster) possess at least one additional row of median spines .

41] Stacked growth pattern of sclerites

0. Absent

1. Present

*Remark*: This character was discussed by Smith and Ortega-Hernández . We include it in this analysis in order to test its influences even if, as mentioned above, its state remains poorly known in most taxa. *Ovatiovermis* possesses stacked posterior claws.

42] Sternites

0. Absent

1. Present

*Remark*: Tardigrades are unique among basal panarthropods in displaying ventral sclerites in addition to dorsal ones. We thus code the presence of sternites as uncertain in Tardigrada, in order to see if sternites and ventral plates can be optimized as homologous through secondary homology.

43] Number of annuli/plicae between lobopods

0. Many (10+)

1. Few (1/8)

*Remark*: This character was formalized by Ramsköld and Chen , although they coded onychophorans as having many annuli. It is possible that they counted all plicae between one leg and another, but this does not correspond to the inter-appendicular space (space between limb insertion and sclerites) in armoured lobopodians. Although state 1 covers a wide range of phenotypes, most taxa are in fact characterized by a rather well constrained series of five inter-appendicular annuli/plicae (e.g. *Onychodictyon* spp., *Hadranax*, *Jianshanpodia*, *Diania*); however *Aysheaia* and *Microdictyon* expand this range with respectively one and eight . Luolishaniids for which this character can be assessed are coded as polymorphic given the dramatic differences in lobopod interspace along the body (see chars. 53-55).

44] Inter-appendicular elongate ventral papillae

1. Absent
2. Present

*Remark*: This character is diagnostic of the genus *Onychodictyon* . We reuse here the wording of Ou *et al*. (they were called “appendicules” in earlier studies [e.g. ).

45] Limb pair number

1. 13 or more
2. 12
3. 11
4. 10
5. 9
6. 8 or fewer

*Remark*: In hallucigeniids, this number includes the two pairs of anterior tentacles.

46] Trunk limb adorned

1. Absent
2. Present

*Remark*: This character codes for the presence of cuticular projections on lobopods (see e.g. M2#9). Setose projections on the limbs are also present in certain tardigrades, e.g. . *Contra* Y#59, we have not found clear evidence of projections on the lobopods of *Onychodictyon ferox* and we consider the spines on *Diania*’s limbs as hypertrophied papillae/plicae only clearly homologous with the spines described in *Antennacanthopodia* (character 48).

47] Lobopod adornment, type

1. Short (base larger than length, length much inferior to limb diameter)
2. Long (slender aspect, subequal or longer than limb diameter)
3. Bi-axial setae (autapomorphy of tardigrades)

*Remark*: We differentiate here the elongate spines (spinules) of *Aysheaia* and luolishaniids from stouter types (e.g. *Diania*).

48] Lobopod spinules paired in a chevron-shaped pattern

0. Absent

1. Present

*Remark*: This character is a luolishaniid synapomorphy ; this study [also see ]. Ma et al 2009 suggested the presence of three or four rows of spinules in *Luolishania*, but two rows seem more in line with the evidence (see also ). All figured specimens show only a pair of lateral spinules along the lobopods, neither the tips of a third or a four pair are preserved, as we should expect, in particular in specimens were the lobopods are twisted and exposed along various angles.

49] Strong antero-posterior tapering of lobopods

1. Absent
2. Present

*Remark*: Taxa such as *Paucipodia*, *Xenusion*, *Diania* and onychophorans lack a strong antero-posterior reduction of trunk limbs otherwise common in lobopodians, dinocaridids and most euarthropods.

50] Exopod

1. Absent
2. Present

*Remark*: We only code here exopod in the euarthropod sense, i.e., as a limb branch borne by a basis alongside an endopod. We therefore do not include the condition of *Aegirocassis* , with respect to which a more detailed dinocaridid phylogeny is needed in order to strengthen the biramous limb origin hypothesis. It has been suggested that exopods among stem arthropods may not be homologous with crustacean exopods , but we refrain from discussing this here, as this does not affect our coding.

51] Limb tip

1. Pad
2. Juxtaposed series of claws
3. Trident of claws
4. Single claw
5. Double claw

*Remark*: As stated in Y#67, the pad/foot of onychophorans is a very peculiar autapomorphy of this group. The arrangement in multiple juxtaposed claws of tardigrades, however, is also observed in *Aysheaia*. The condition in *Luolishania* has been the subject of debate e.g. , and we code it here as uncertain. The coding of multiple terminal claws in *Luolishania*, however, has a strong impact on the likelihood-based phylogenetic reconstruction, which is discussed above and in the main text. The “trident” of claws is here autapomorphic of *Olenoides* . For taxa having single claws exclusively in posterior limbs, we use a separate character (char. 58).

**POSTERIOR SECTION**

52] Lobopodous trunk posterior termination type

1. Vestigial (“truncated”)
2. Well-developed

*Remark*: The posterior termination of the lobopodian trunk is either truncated (i.e. reduced) or protruding as a portion of the trunk subequal to the penultimate interappendicular trunk segment. The claim (e.g. Yang *et al*. char. 75) that there is a posterior cuticular extension of the trunk in *Collinsium* is unsubstantiated to us given their photographic documentation, and we coded this taxon as uncertain, as is the alleged trunk extension of *Luolishania* . In the case of *Collinsium*, this seems to be but a breakage in the fossil, and the annuli of the left terminal lobopod are in fact still visible on what is designated as a trunk extension; in *Luolishania* , it is clear that the last pair of lobopods can only attach to this portion of the trunk, which shows rather well the truncated posterior end of the body. The importance of this character was emphasized by Ramsköld and Chen (, R2#9), but we revised their coding to include hallucigeniids (see e.g. *Hallucigenia*, , *contra* Y#75) and luolishaniids, as well as tardigrades. The condition in *Microdictyon* is similar to hallucigeniids, in which the body ends abruptly with the last pair of lobopods, with no trace of further trunk projection.

53] Interspace between lobopods in the anterior section of body

1. Subequal
2. Increasing posteriad

*Remark*: This character and the following refer to the pattern discussed by Ma et al. (2009) and Yang et al. (2016) in the context of luolishaniids. We separate anterior and posterior differentiations in lobopod interspace length as we noticed that certain taxa displayed one but not the other.

54] Interspace between lobopods in the posterior section of body

1. Subequal
2. Increasing anteriad

55] Number of limb pairs affected by the reduction of posterior interspace

1. Two (penultimate and last)
2. More than two

56] Posteriormost set of differentiated limbs

1. Absent
2. Present

*Remark*: See M2#35 and Y#42. This character codes for the morphological differentiation of posteriormost lobopods (see char. 58).

57] Posteriormost set of differentiated limbs, type

1. Stout, clawed (anchoring)
2. Claw lost, flap or fan-like (tailpiece)

*Remark*: The first state refers here in particular to the thickening, and in the general the shortening, of posterior lobopods compared to more anterior limbs, as is characteristic of luolishaniids, *Luolishania* excepted. *Ovatiovermis* represents the most extreme example of this condition. Often called “tail fans” in dinocaridids (e.g. ), sets of differentiated terminal limbs forming a tailpiece are also tentatively coded as present in *Jianshanopodia*, although in this case their morphology is mostly that of reduced trunk limbs.

58] Single claws on posteriormost lobopodous limbs

1. Absent
2. Present

*Remark*: Yang *et al.* 2015 noted that single claws seem to characterize the posterior limbs of a number of luolishaniids and hallucigeniids. We reevaluated the evidence and largely concur with the validity of this character, in spite of the taphonomic uncertainty related to the exact count of claws. The evidence is particularly strong in *Hallucigenia sparsa* , with dozens of specimens corroborating the observation. We also think that the evidence is sound in *Ovatiovermis*, as the holotype and paratype perfectly preserve several of their posteriormost claws (Figs. 1 and 2).

59] Posteriormost claws pointing anteriad

0. Absent

1. Present

*Remark*: As discussed by various authors (Y#80), a number (to us, a majority) of lobopodians share with tardigrades an anterior orientation of the posteriormost claws. It is especially clear and consistent across specimens of *Aysheaia* and *Hallucigenia* , rejecting a taphonomic origin of this condition. This condition has also been recognized in *Microdictyon* .

**
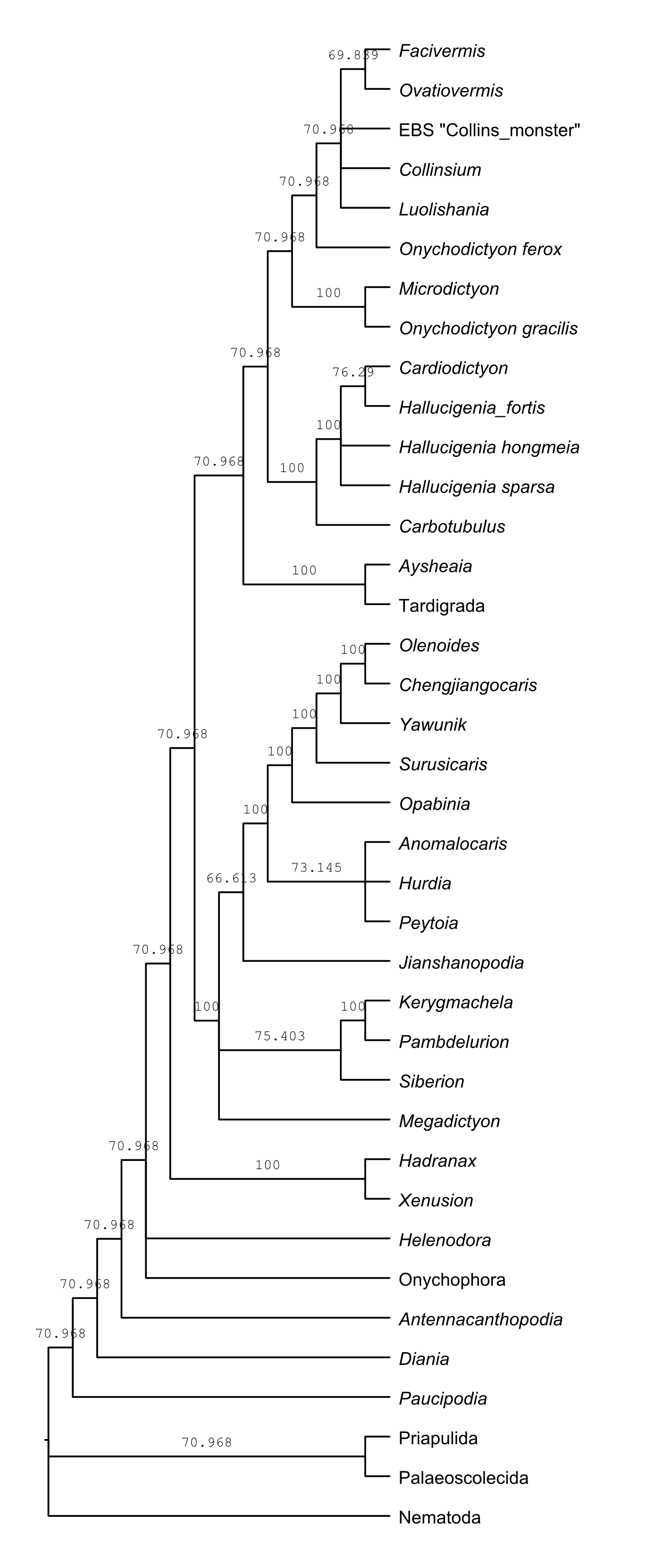
**

**Figure S1**. **Majority rule consensus of parsimony (from 1240 MPT trees of 124 steps). Frequencies of occurrence written above branches. By comparison, the Bayesian consensus tree is 133 steps when optimized under parsimony.**

**Disparity analyses**

**Reanalysis of the data in Yang *et al*. 2015 :** When running a PCoA with the dataset published by Yang *et al*. 2015 using the dissimilarity function *dist*, we obtain a roughly similar result (**Figure S2**) to their published morphospace. (We have kept taxa, such as *Aysheaia* and *Onychodictyon ferox*, which may explain some of the discrepancies). It appears, however, that *Acinocricus* is largely responsible for such a dramatic stretch of the luolishaniid morphospace (**Figure S2**). In fact, this taxon, along with *Ortenotubulus*, the Collins’ monsters, including the Emu Bay Shale form, and *Hallucigenia hongmeia*, account for the greatest number of uncertainties. As we realized during our own analysis (see below), those unknown character states have most certainly distorted the ordination and, in this case, greatly inflated luolishaniid disparity. More importantly, *dist* has been designed to calculate dissimilarities for numerical data, and not factorial data as is the case with the morphological matrices considered here. Consequently, the resulting graphical representation, which sets aside luolishaniids from other lobopodians on axes 1 and 2, is unreliable (**Figure S2)**. When computing ordinations based on dissimilarities obtained with *daisy*, with characters designated as factorial variables, we obtain a representation much more similar to the morphospace presented in this study (**Figure S2**), with three distinct clusters of taxa corresponding to the luolishaniids, hallucigeniids and onychophorans. (We have here left *Diania* outside of the onychophoran space).


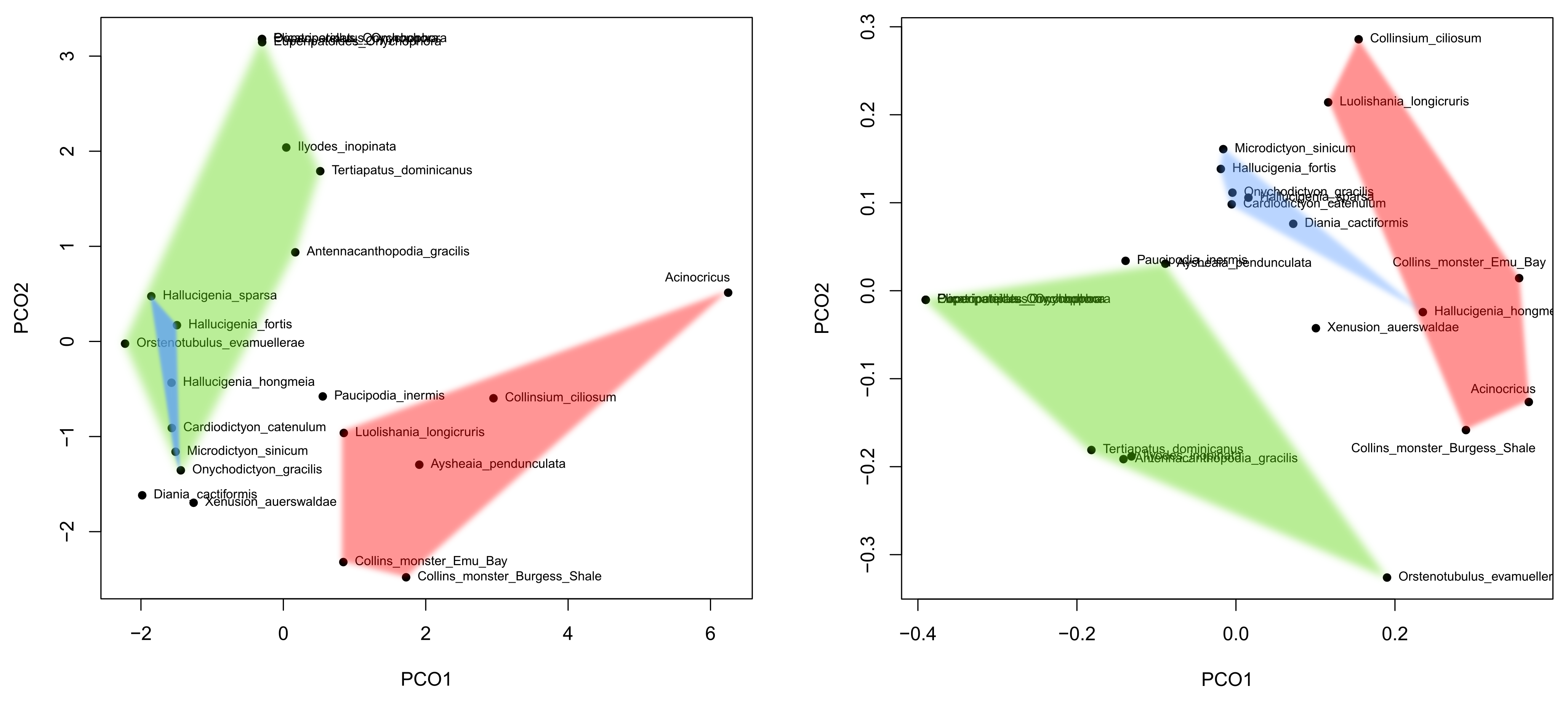


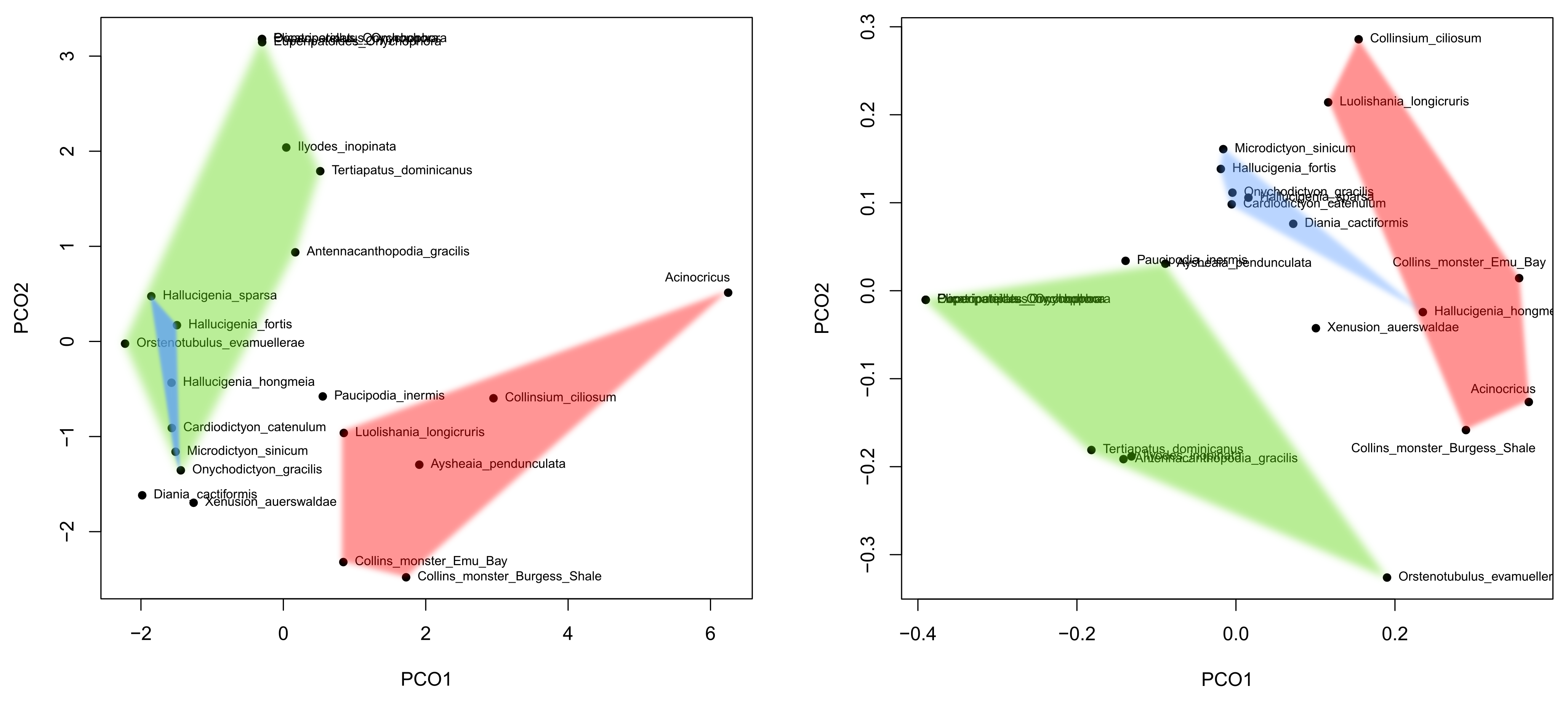


**Figure S2**. PCoA of the unaltered Yang *et al*. 2015 data using dissimilarities calculated with *dist* (top) and *daisy* (bottom). Red (to the right) corresponds to luolishaniids, green to onychophorans (to the left) and blue to hallucigeniids (smaller areas to the left and right, respectively).

**Results with our own data set**: We used here the method described in a previous study and draw on earlier works on morphological disparity using fossil data . Deviations from our original procedure are detailed below. In particular, the characterization of the morphospace of interest took place in several steps. Throughout those steps, in order to preserve the signal coded as plesiomorphies within the data set, relevant taxa were duplicated and each duplicate was coded with a state variation. Because this artificial inflation of the number of taxa can affect the calculation of disparity metrics, especially when using resampling methods, the PCoA was secondarily corrected by averaging coordinate values between duplicates so as to obtain a single data point per taxon. Inapplicable entries were manually converted into question marks, as *daisy* (contrarily to *dist*) treats “-” as an additional factor value. Each step was accompanied by an analysis of the number of significant axes and a k-means clustering analysis with estimation of the optimal number of groups based on the Calinski criterion. Characters were also removed between steps as they were becoming non-informative/constant with the removal of taxa, although we kept characters becoming autapomorphic.

Step 1 **(Figure S3)**: We built the dissimilarity matrix with the entire dataset used for the phylogenetic analysis. The first three axes explained a greater percentage of variation than expected from the Broken Stick model. K-means clustering was optimal for two groups, segregating dinocaridids and euarthropods from the rest of the sample. The morphological gap was particularly noticeable on the graphical representation of the morphospace using axes 1 and 2.

Step 2: As arthropod taxa and their idiosyncratic characters represent a distinct sector of the morphospace and can arguably distort the ordination of lobopodians, we removed them from the data set, along with the outgroup (Nematoda, Priapulida, Palaeoscolecida), which also clustered outside the lobopodian region. The first three axes explained a greater percentage of variation than expected from the Broken Stick model **(Figure S4)**. K-means clustering was optimal for two groups, this time segregating hallucigeniids and luolishaniids from all other lobopodians. On the graphical reconstructions, the Emu Bay Shale “Collins’ monster,” *Hadranax* and *Hallucigenia hongmeia* strikingly stood out as outliers on the highest values of the second axis of principal coordinates. The only common characteristic of these taxa beyond their general lobopodian affinity is their greater number of associated missing entries compared to others. While certain taxa (e.g. *Facivermis*) suffered from an inflation of question marks after the conversion of inapplicable states, the EBS “Collins’ monster,” *Hadranax* and *Hallucigenia hongmeia* directly inherited this uncertain coding from the data set, in large part because of missing information on their anterior and posterior anatomies. Leaving these taxa would have therefore artificially expanded the morphospace of the relevant groups, and especially that of luolishaniids. *Kerygmachela* and its relatives are similarly characterized by higher numbers of missing entries, although this did not seem to affect their clustering. Because our phylogenetic analyses suggest that these taxa are more closely related to arthropods and because their morphospace does not overlap with that of other lobopodians, we also excluded them from the final analysis. We kept other taxa with high levels of uncertainties (*Xenusion* and *Carbotubulus*) because they were not grouped with the main outliers, but it must be kept in mind that they may contribute to slightly distort estimations of disparity for hallucigeniids and onychophorans.

Step 3 **(Figure S5)**: The PCoA of our corrected data set has four axes explaining a greater percentage of variation than expected from the Broken Stick model. The result of the k-means Calinski test finds two optimal clusters: a hallucigeniid-luolishaniid group and a tardigrade/onychophoran group. Those clusters correspond to our Bayesian tree with the exception of *Carbotubulus* and *Facivermis* being interpolated by the k-means with the tardigrade-onychophoran grade. The grouping of hallucigeniids with luolishaniids without *Carbotubulus* and *Facivermis* is supported by a neighbour-joining analysis of the dissimilarity matrix (function njs from the R package *ape*, designed to work with matrices containing missing data). For disparity metrics, we used sum of ranges and hypervolume (based on simulated data), but not sum of variances, as variance is dependent on taxon number (and therefore morphospace “filling”), which could be dramatically sensitive when dealing with groups composed of a handful of taxa. We compared luolishaniids sensu stricto (excluding *Facivermis*), hallucigeniids sensu lato (including *Microdictyon*, given the consistency between our cladogram and the k-means test) and strict onychophoran-like taxa (*Antennacanthopodia*, *Helenodora* and Onychophora), leaving the “tardigrade grade” only as part of the calculation for all taxa. Our jackknifing of sum of ranges showed that the very limited taxa sample was likely underestimating the disparity for luolishaniids and onychophoran-like taxa and suggested that hallucigeniids and luolishaniids covered a greater range of morphospace than onychophoran-like taxa. Due to the limited sampling, permutation tests do not find a significant difference between the sums of ranges of any group, based on the original data only. However, while the simulated data does not show a significant difference in sums of ranges between luolishaniids and hallucigeniids, sums of ranges between each of these groups and onychophoran-like taxa are found to differ (Mann-Whitney U test, p-values 0.31 and <10^-3^ respectively). Hypervolume results provide a different picture **(Figure S6),** with hallucigeniids occupying a greater amount of morphospace than other groups. with sets of simulated data points significantly different from one another (N= 57776 (luolishaniids); N= 99072 (hallucigeniids); N= 61340 (onychophorans); Mann-Whitney U test, p-values <10^-3^) but with negligeble overlap based on the Sorensen index (luolishaniids/onychophoran-like= 0; luolishaniids/hallucigeniids= 0.03395289; onychophoran-like/hallucigeniids= 0.05916587). Centroids for all three groups are roughly equidistant (Euclidean distances: luolishaniids/onychophorans= 0.6489781; luolishaniids/hallucigeniids= 0.5178908; onychophorans/hallucigeniids= 0.5182448). For the calculation of hypervolumes, we used a bandwith of 0.2, based on an average of estimated bandwidth (Silverman estimator) on the first four PCoA axes. Finally, we tested the addition of two autapomorphic characters representing slime papillae and jaws for Onychophora in order to see their impact on the analysis, but neither the morphospace nor the disparity metrics were changed.


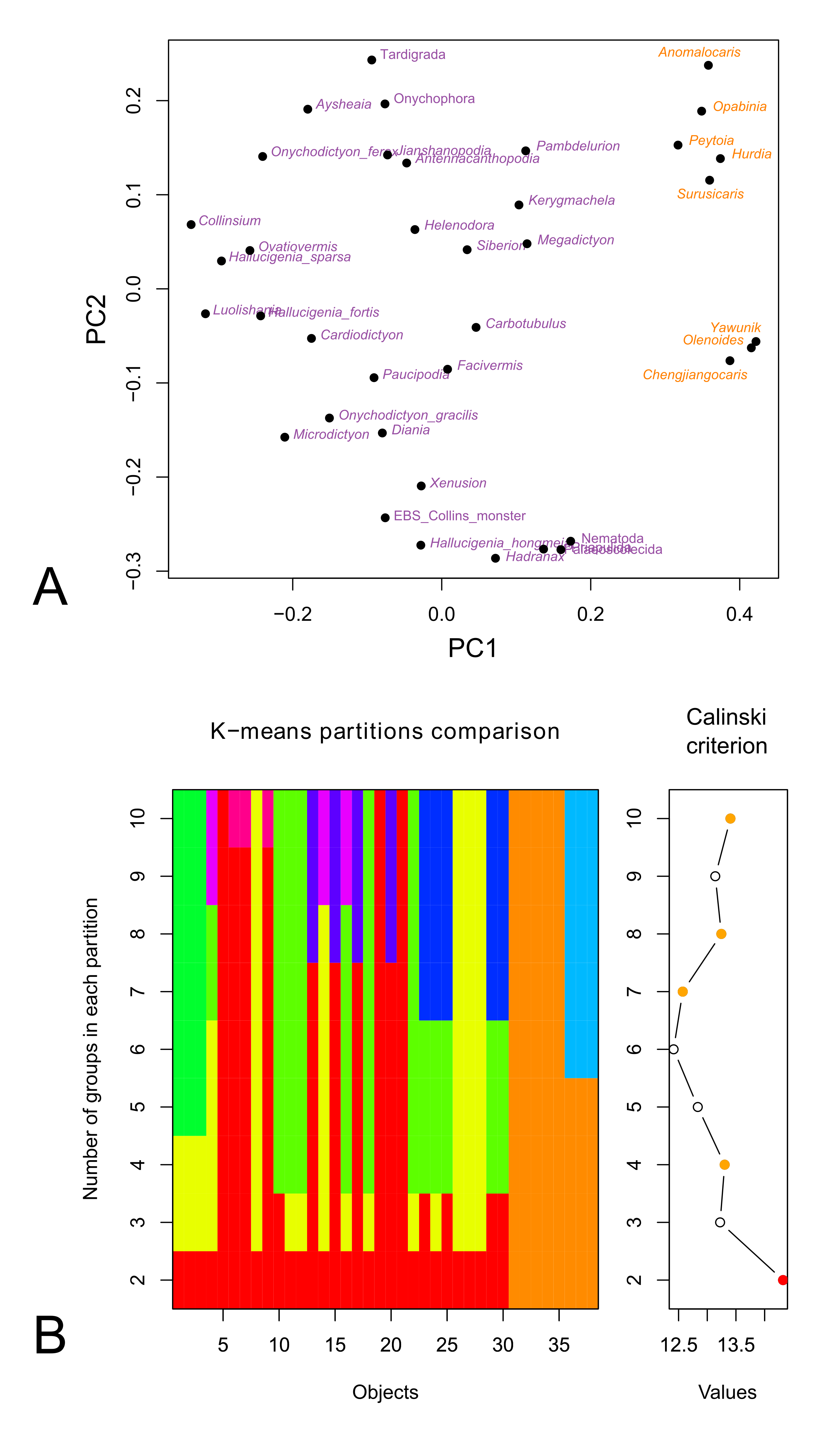


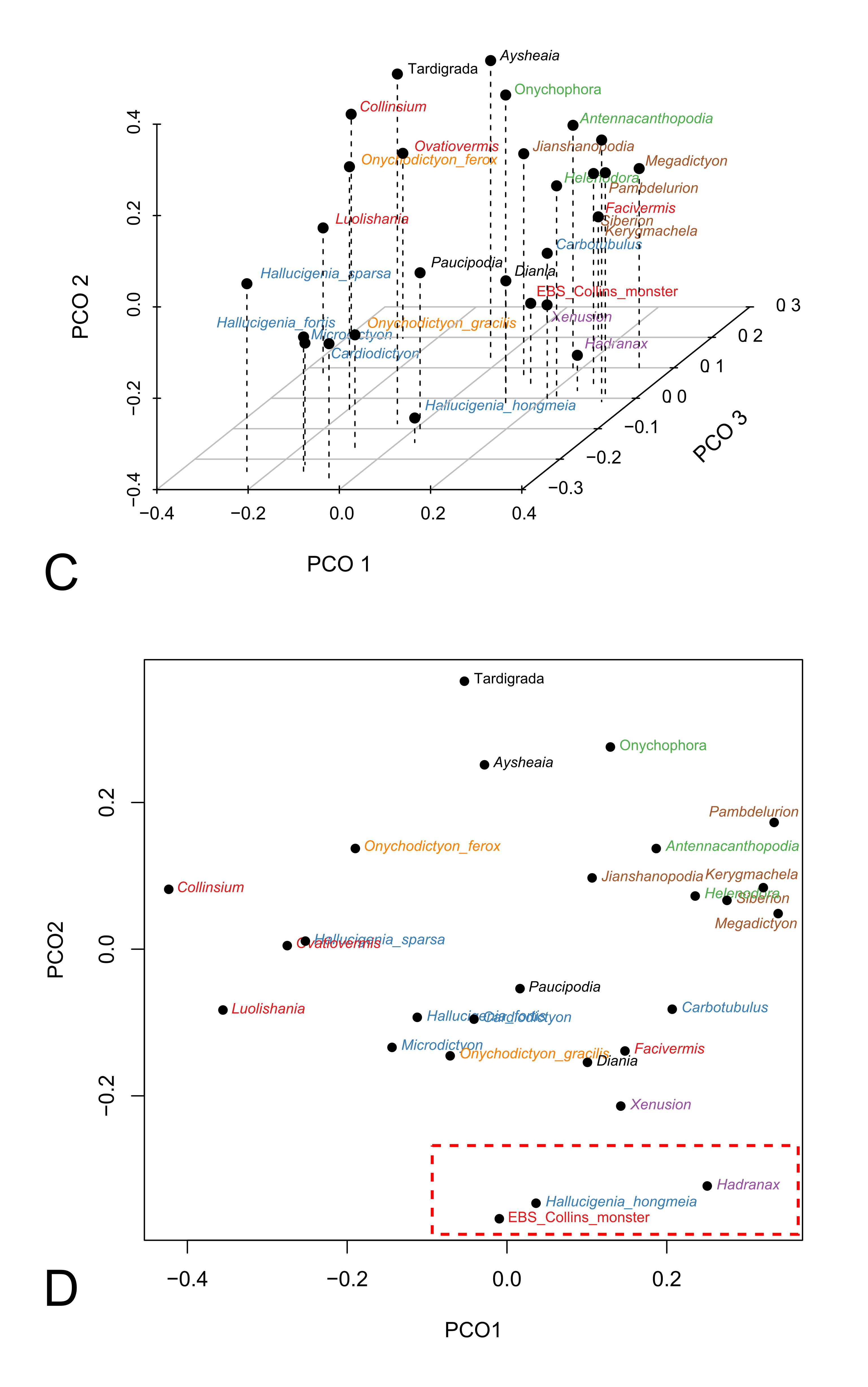


**Figure S3**. Preliminary steps in the reconstruction of the lobopodian morphospace. (A). PCoA for the entire set of taxa used for the phylogeny with colouring based on (B) k-means clustering with optimal Calinski criterion of 2 groups. Arthropods are discriminated from other taxa. (C) and (D). Morphospace with arthropods and outgroup excluded, colouring based on cladistic results. *Hallucigenia hongmeia*, *Hadranax* and the Emu Bay Shale Collins’ monster form an outlying cluster towards the maximal values of axis 2 (see also **Table S2**).

*
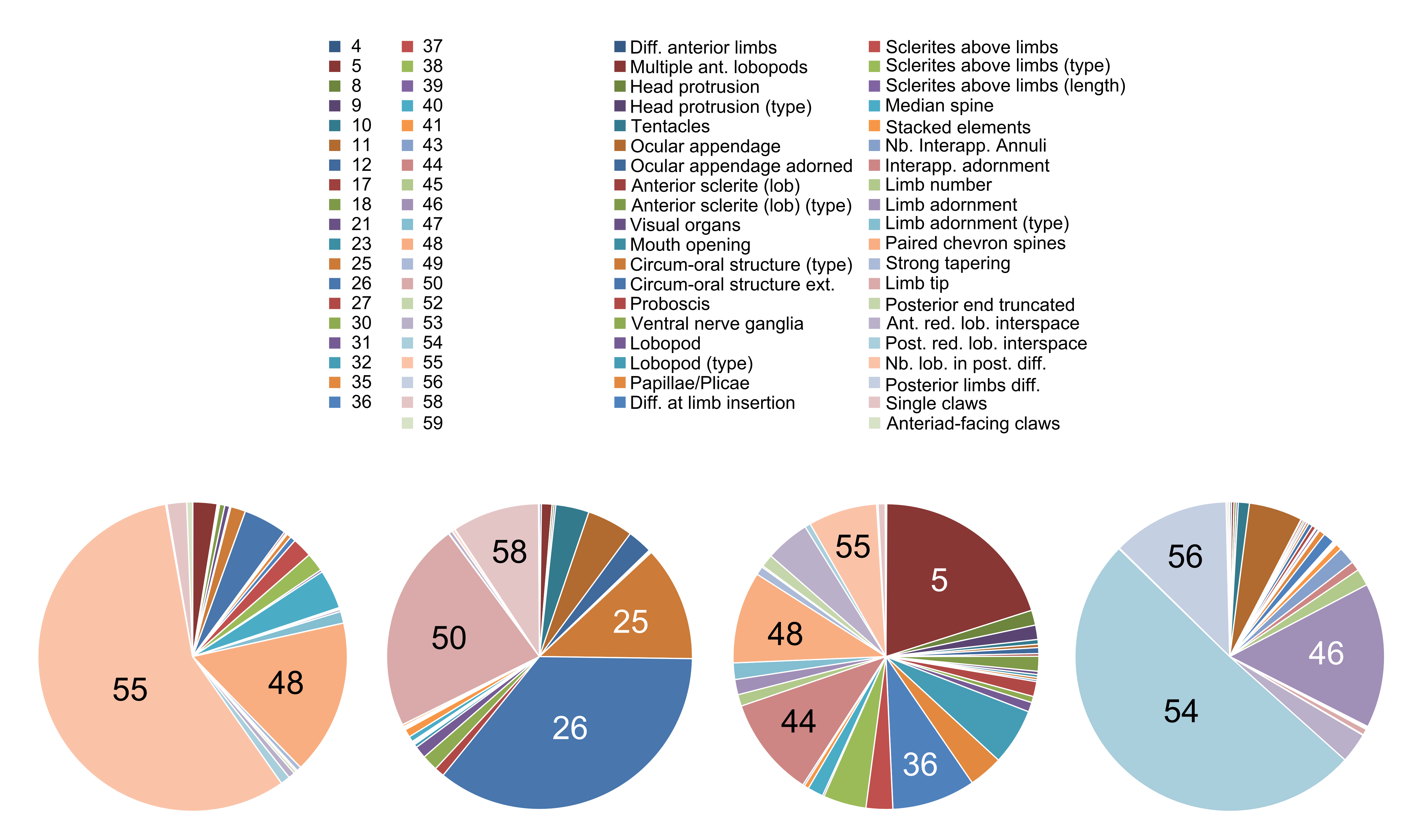
*

**Figure S4.** Relative influence for each character on the first four axes (left to right) of the PCoA (p-values from **Table S4**). Percentages of variation explained: PC1: 28.82, PC2: 19.46, PC3: 16.20, PC4: 10.88.

|  | Before conversion | After conversion |
| --- | --- | --- |
| *Facivermis* | 27 | 10 |
| *Paucipodia* | 18 | 3 |
| *Cardiodictyon* | 14 | 7 |
| *Hallucigenia fortis* | 12 | 7 |
| *Hallucigenia hongmeia* | 28 | 25 |
| *Hallucigenia sparsa* | 7 | 1 |
| *Jianshanopodia* | 14 | 7 |
| *Kerygmachela* | 25 | 18 |
| *Pambdelurion* | 24 | 16 |
| *Ovatiovermis* | 9 | 2 |
| EBS "Collins monster" | 26 | 25 |
| *Collinsium* | 3 | 2 |
| *Carbotubulus* | 27 | 22 |
| *Luolishania* | 9 | 7 |
| *Megadictyon* | 27 | 23 |
| *Microdictyon* | 15 | 5 |
| *Onychodictyon ferox* | 9 | 3 |
| *Onychodictyon gracilis* | 18 | 7 |
| *Siberion* | 23 | 18 |
| *Antennacanthopodia* | 18 | 9 |
| *Helenodora* | 20 | 10 |
| Onychophora | 10 | 0 |
| *Diania* | 22 | 9 |
| *Hadranax* | 34 | 28 |
| *Xenusion* | 26 | 18 |
| *Aysheaia* | 10 | 3 |
| Tardigrada | 11 | 0 |

**Table S2. Number of question marks coded for lobopodian taxa before and after conversion of inapplicable entries.**


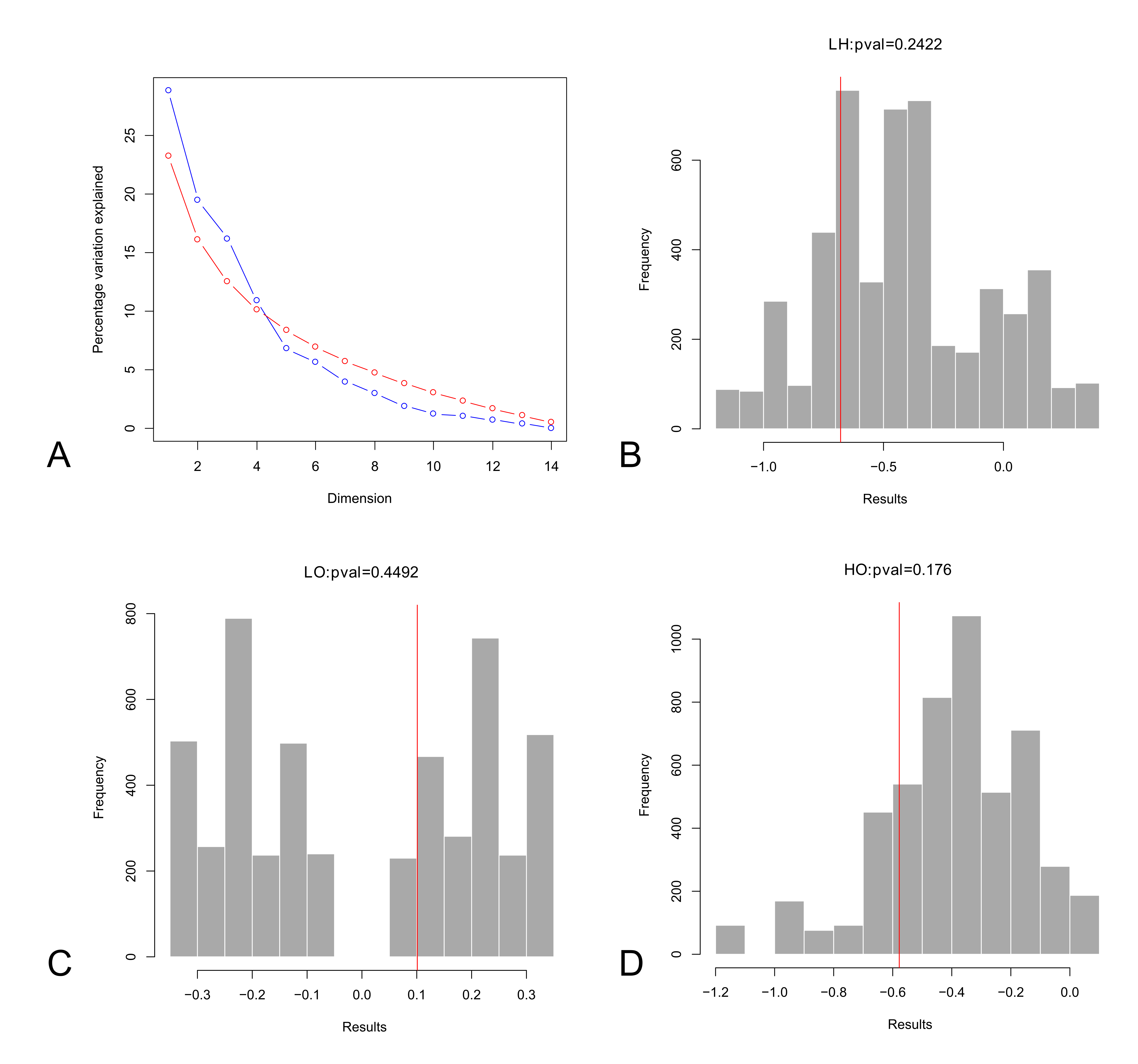


**Figure S5**. Descriptive tests of the corrected dataset (see Step 3 above). (A) Percentage of total variation explained by each axis (blue) compared to the Broken Stick model (red). (B) Permutation test results for the sum of ranges (observed value) between luolishaniids and hallucigeniids (p-value: 0.242). (C) Permutation test results for the sum of ranges between luolishaniids and panonychophorans (p-value: 0.449). (D) Permutation test results for the sum of ranges between hallucigeniids and panonychophorans (p-value: 0.176).

**
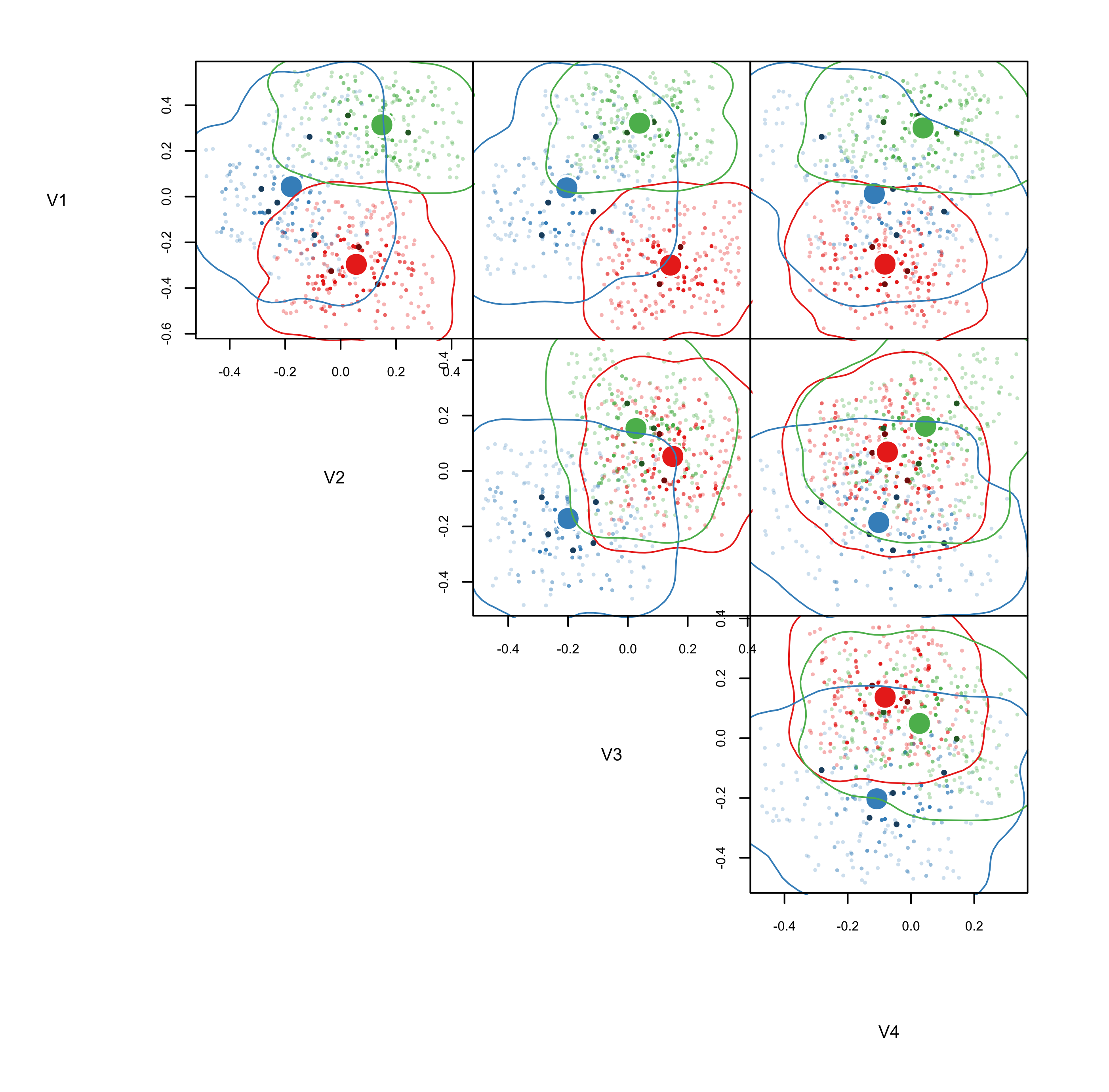
**

**Figure S6**. Hypervolume projections on the first four axes of the morphospace. Red: luolishaniids; blue: hallucigeniids; green: panonychophorans. Small dark dots are data points; large dots are respective cluster centroids. Contour lines are only for ease of reading and are drawn based on a five percent quantile threshold of projected data using a two-dimensional kernel density estimator (*kde2d*, *R* package *MASS*).

**Relative influence of characters:** We evaluated the influence of the morphological characters on the morphospace using two different approaches. The first test relied on the function *envfit* from the *R* package *vegan*, which runs linear regression analyses between factors and computes a p-value from permutation tests. This constitutes an indicator of character influence on all selected axes, here 1 to 4. While most characters have a significant influence on the morphospace, only 13 are found to be highly significant (Table S2, in bold). These characters are also critical for the reconstruction of phylogenetic relationships between lobopodians and arguably synthesize the main aspects of morphological evolution within this group, as developed in the main text of this study. Note however that important characters such as hallucigeniid tentacles and luolishaniid posterior stout limbs have lower significance levels, suggesting that they may have secondarily contributed to shaping the morphospace. Indeed, *Microdictyon*, which lacks the typical hallucigeniid tentacles, is close to or part of the hallucigeniid morphospace, while *Luolishania* itself lacks the conspicuously stouter poster limbs found in *Ovatiovermis* and *Collinsium*.

Our second approach follows Aria and Caron and is based on the calculation of chi-square statistics on arbitrarily subdivided PC axes, a method initially developed by Foote (1995). This notably allows the investigation of the relative character influence on each dimension of the morphospace. According to this method, the posterior configuration of the trunk in having either one or more reduced lobopod interspace(s) (55) shapes most of the ordination on axis 1, which indeed reveals itself very effective in segregating luolishaniids from hallucigeniids. This character also contributes to the separation of the *Onychodictyon* taxa as previously discussed. The “paired chevron spinules” (49) supports this character in shaping the PCO 1. Axis 2 is dominated by characters related to the mouth, as well as by the type of main lobopod claw and, importantly, the presence of single claws on posterior lobopods. Axis 3 is more complex, likely contributing to a large number of smaller adjustments between taxa in the morphospace, but puts forward modifications of the trunk as well as the recruitment of several anterior lobopods towards differentiation (5) as influential characters. Finally, axis 4 is dominated by the sovereign character of character 55, which significantly contributes to separating onychophoran-like taxa from the others, complemented by the differentiation of posterior limbs (in luolishaniids) and limb adornment—a character also strengthening the clustering of luolishaniids.

| Nb# | Character | R squared | P-value | Sig. level |
| --- | --- | --- | --- | --- |
| 4 | Diff. anterior limbs | 0.1549 | 0.091908 | . |
| 5 | Multiple ant. limbs diff. | 0.3284 | 0.003996 | ** |
| 8 | Head protrusion | 0.1343 | 0.043956 | * |
| 9 | Head protrusion (type) | 0.141 | 0.166833 |  |
| 10 | Postoral tentacles | 0.3043 | 0.002997 | ** |
| 11 | Ocular appendage | 0.2748 | 0.00999 | ** |
| 12 | Ocular appendage adorned | 0.3263 | 0.002997 | ** |
| 17 | Anterior sclerite (lob) | 0.1753 | 0.016983 | * |
| 18 | **Anterior sclerite (lob) (type)** | 0.3057 | 0.000999 | *** |
| 21 | Visual organs | 0.2382 | 0.022977 | * |
| 23 | **Mouth opening** | 0.2555 | 0.000999 | *** |
| 25 | **Circum-oral structure (type)** | 0.5838 | 0.000999 | *** |
| 26 | **Circum-oral structure ext.** | 0.5827 | 0.000999 | *** |
| 27 | Proboscis | 0.2686 | 0.006993 | ** |
| 30 | Ventral nerve ganglia | 0.2085 | 0.00999 | ** |
| 31 | Lobopod | 0.1194 | 0.040959 | * |
| 32 | **Lobopod (type)** | 0.393 | 0.000999 | *** |
| 35 | Papillae/Plicae | 0.0823 | 0.455544 |  |
| 36 | **Diff. at limb insertion** | 0.3938 | 0.000999 | *** |
| 37 | **Sclerites above limbs** | 0.4385 | 0.000999 | *** |
| 38 | Sclerites above limbs (type) | 0.2873 | 0.00999 | ** |
| 39 | Sclerites above limbs (length) | 0.3131 | 0.002997 | ** |
| 40 | **Median spine** | 0.4338 | 0.000999 | *** |
| 41 | Stacked elements | 0.2334 | 0.016983 | * |
| 43 | Nb. Interapp. Annuli | 0.1434 | 0.174825 |  |
| 44 | Interapp. Adornment | 0.1057 | 0.328671 |  |
| 45 | Limb number | 0.2073 | 0.769231 |  |
| 46 | Limb adornment | 0.1347 | 0.136863 |  |
| 47 | Limb adornment (type) | 0.3152 | 0.008991 | ** |
| 48 | Paired chevron spinules | 0.2619 | 0.006993 | ** |
| 49 | Strong tapering | 0.2995 | 0.004995 | ** |
| 50 | **Limb tip** | 0.4258 | 0.000999 | *** |
| 52 | **Posterior end truncated** | 0.4124 | 0.000999 | *** |
| 53 | Ant. reduction of lob. inters. | 0.3684 | 0.001998 | ** |
| 54 | Post. reduction of lob. inters. | 0.3502 | 0.001998 | ** |
| 55 | **Nb. lob. in post. differentiation** | 0.4189 | 0.000999 | *** |
| 56 | Posterior limbs diff. (type) | 0.0689 | 0.648352 |  |
| 58 | **Single claws** | 0.6366 | 0.000999 | *** |
| 59 | **Anteriad-facing claws** | 0.4665 | 0.000999 | *** |

**Table S3**. Signif. codes: 0 ‘***’ 0.001 ‘**’ 0.01 ‘*’ 0.05 ‘.’ 0.1 ‘ ’ 1

|  | PCO1 | PCO2 | PCO3 | PCO4 |
| --- | --- | --- | --- | --- |
| Diff. anterior limbs | 0.6467658547 | 0.0998788163 | 0.5603932736 | 0.2228208477 |
| Multiple ant. limbs diff. | 0.0047778574 | 0.0179426948 | 0.0024318383 | 0.1527194885 |
| Head protrusion | 0.3237076063 | 0.0959253318 | 0.0307432147 | 0.1757325169 |
| Head protrusion (type) | 0.4517698671 | 0.1074816074 | 0.031951837 | 0.1929661028 |
| Postoral tentacles | 0.585951308 | 0.0056693572 | 0.0988332385 | 0.0347347332 |
| Ocular appendage | 0.9837148944 | 0.0041027965 | 0.13735684 | 0.0072319595 |
| Ocular appendage adorned | 0.5555347735 | 0.0075982105 | 0.0762892671 | 0.5230503097 |
| Anterior sclerite (lob) | 0.0896531146 | 0.5148562322 | 0.1773092496 | 0.2098959568 |
| Anterior sclerite (lob) (type) | 0.0224557532 | 0.1537950179 | 0.0308216564 | 0.2357867272 |
| Visual organs | 0.0238537613 | 0.3354928936 | 0.1437330037 | 0.2117791372 |
| Mouth opening | 0.086307415 | 0.2560060178 | 0.1637145779 | 0.8374808827 |
| Circum-oral structure (type) | 0.0077758022 | 0.001646939 | 0.1865330887 | 0.1748493731 |
| Circum-oral structure ext. | 0.0026522088 | 0.0005617112 | 0.2471127064 | 0.0948923459 |
| Proboscis | 0.0622981145 | 0.0191985841 | 0.0309072321 | 0.1010159837 |
| Ventral nerve ganglia | 0.5477125243 | 0.011818514 | 0.0740991154 | 0.3002254747 |
| Lobopod | 0.4305185012 | 0.0154210667 | 0.04843365 | 0.1712996928 |
| Lobopod (type) | 0.0969760947 | 0.0577855277 | 0.0081547785 | 0.3002254747 |
| Papillae/Plicae | 0.025854332 | 0.1756867797 | 0.0134555274 | 0.063721403 |
| Diff. at limb insertion | 0.0214699945 | 0.1400115803 | 0.0055214495 | 0.0346839963 |
| Sclerites above limbs | 0.0058179444 | 0.2868830265 | 0.0171952184 | 0.3917040941 |
| Sclerites above limbs (type) | 0.0061712845 | 0.5080934146 | 0.0107239279 | 0.4943974207 |
| Sclerites above limbs (length) | 0.0536547176 | 0.6850267949 | 0.2863862766 | 0.2907085631 |
| Median spine | 0.0029500225 | 0.0346839963 | 0.0293028366 | 0.3199673405 |
| Stacked elements | 0.2364009947 | 0.02369122 | 0.0999983813 | 0.0621283426 |
| Nb. Interapp. Annuli | 0.5379827879 | 0.3003714791 | 0.2580612347 | 0.0224130708 |
| Interapp. Adornment | 0.1487906294 | 0.1700902812 | 0.0045872971 | 0.0408226301 |
| Limb number | 0.2072213221 | 0.227446642 | 0.0395339928 | 0.022867404 |
| Limb adornment | 0.0559987807 | 0.4380637585 | 0.0302708581 | 0.0026607344 |
| Limb adornment (type) | 0.0098794193 | 0.3226240199 | 0.0279258749 | 0.3331105168 |
| Paired chevron spinules | 0.0007465013 | 0.0970912349 | 0.0050077428 | 0.3520393953 |
| Strong tapering | 0.0256651351 | 0.9031274478 | 0.0539906899 | 0.4031386697 |
| Limb tip | 0.0744151027 | 0.0009015981 | 0.3207352607 | 0.0642185932 |
| Posterior end truncated | 0.0471866984 | 0.3268811126 | 0.0364648222 | 0.6447429774 |
| Ant. reduction of lob. inters. | 0.0180060162 | 0.0536072394 | 0.0103704234 | 0.0128548409 |
| Post. reduction of lob. inters. | 0.0121401919 | 0.3877850079 | 0.0803817022 | 0.0007964786 |
| Nb. lob. in post. differentiation | 0.0002124062 | 0.0846333377 | 0.0066847389 | 0.583631113 |
| Posterior limbs diff. (type) | 0.0853820751 | 0.2175943096 | 0.3286880466 | 0.0032919139 |
| Single claws | 0.0058804101 | 0.0021691335 | 0.0642269307 | 0.2228208477 |
| Anteriad-facing claws | 0.0197987718 | 0.2201947765 | 0.6797778157 | 0.1986525021 |

**Table S4.** P-values of chi-square tests calculated for each character on the first four axes of the PCoA.

**Supplementary references**

1. Ronquist F, Teslenko M, van der Mark P, Ayres DL, Darling A, Hohna S, Larget B, Liu L, Suchard MA, Huelsenbeck JP: **MrBayes 3.2: efficient Bayesian phylogenetic inference and model choice across a large model space**. *Syst Biol* 2012, **61**(3):539-542.

2. Swofford DL: **PAUP: Phylogenetic Analysis Using Parsimony (*and Other Methods), ver. 4.0a147**. In*.* Sunderland, Massachusetts: Sinauer Associates; 2003.

3. Lewis PO: **A likelihood approach to estimating phylogeny from discrete morphological character data**. *Systematic Biology* 2001, **50**(913-925).

4. Aria C, Caron J-B, Gaines R: **A large new leanchoiliid from the Burgess Shale and the influence of inapplicable states on stem arthropod phylogeny**. *Palaeontology* 2015, **58**(4):629-660.

5. Ramsköld L, Chen JY: **Cambrian lobopodians: morphology and phylogeny**. In: *Arthropod fossils and phylogeny.* Edited by Edgecombe GD, vol. 29. New York: Columbia University Press; 1998: 107-150.

6. Liu J, Steiner M, Dunlop JA, Keupp H, Shu D, Ou Q, Han J, Zhang Z, Zhang X: **An armoured Cambrian lobopodian from China with arthropod-like appendages**. *Nature* 2011, **470**(7335):526-530.

7. Ma X, Hou X, Bergström J: **Morphology of *Luolishania longicruris* (Lower Cambrian, Chengjiang Lagerstätte, SW China) and the phylogenetic relationships within lobopodians**. *Arthropod Structure & Development* 2009, **38**(4):271-291.

8. Ma X, Edgecombe GD, Legg DA, Hou X: **The morphology and phylogenetic position of the Cambrian lobopodian *Diania cactiformis***. *Journal of Systematic Palaeontology* 2014, **12**(4):445-457.

9. Smith MR, Ortega-Hernández J: ***Hallucigenia*'s onychophoran-like claws and the case for Tactopoda**. *Nature* 2014, **514**(7522):363-366.

10. Yang J, Ortega-Hernández J, Gerber S, Butterfield NJ, Hou J-b, Lan T, Zhang X-g: **A superarmored lobopodian from the Cambrian of China and early disparity in the evolution of Onychophora**. *Proceedings of the National Academy of Sciences* 2015, **112**(28):8678-8683.

11. Smith MR, Caron J-B: ***Hallucigenia*'s head and the pharyngeal armature of early ecdysozoans**. *Nature* 2015, **523**(7558):75-78.

12. Murdock DJE, Gabbott SE, Purnell MA: **The impact of taphonomic data on phylogenetic resolution: *Helenodora inopinata* (Carboniferous, Mazon Creek Lagerstätte) and the onychophoran stem lineage**. *BMC Evolutionary Biology* 2016, **16**(1):1-14.

13. Mayer G, Martin C, Rüdiger J, Kauschke S, Stevenson PA, Poprawa I, Hohberg K, Schill RO, Pflüger H-J, Schlegel M: **Selective neuronal staining in tardigrades and onychophorans provides insights into the evolution of segmental ganglia in panarthropods**. *BMC Evolutionary Biology* 2013, **13**(1):1-16.

14. Wiens JJ: **Missing Data, Incomplete Taxa, and Phylogenetic Accuracy**. *Systematic Biology* 2003, **52**(4):528-538.

15. Prevosti FJ, Chemisquy MA: **The impact of missing data on real morphological phylogenies: influence of the number and distribution of missing entries**. *Cladistics* 2010, **26**(3):326-339.

16. Conway Morris S: **A new metazoan from the Cambrian Burgess Shale of British Columbia**. *Palaeontology* 1977, **20**(3):623-640.

17. Ramsköld L: **Homologies in Cambrian Onychophora**. *Lethaia* 1992, **25**:443-460.

18. Daley AC, Budd GE, Caron J-B, Edgecombe GD, Collins D: **The Burgess Shale anomalocaridid *Hurdia* and its significance for early euarthropod evolution**. *Science* 2009, **323**:1597-1600.

19. Ma X, Edgecombe GD, Legg DA, Hou X: **The morphology and phylogenetic position of the Cambrian lobopodian Diania cactiformis**. *J Syst Palaeontol* 2013, **12**.

20. Budd GE: **Tardigrades as ‘stem-group arthropods’: the evidence from the Cambrian fauna**. *Zoologischer Anzeiger - A Journal of Comparative Zoology* 2001, **240**(3–4):265-279.

21. Hou XG, Aldridge RJ, Bergström J, Siveter DJ, Siveter DJ, Feng XH: **The Cambrian fossils of Chengjiang, China: the flowering of early animal life**. Oxford: Blackwell; 2004.

22. Harvey THP, Dong X, Donoghue PCJ: **Are palaeoscolecids ancestral ecdysozoans?** *Evolution & Development* 2010, **12**(2):177-200.

23. Scholtz G: **The Articulata hypothesis - or what is a segment?** *Organisms Diversity & Evolution* 2002, **2**(3):197-215.

24. Oliveira IS, Lacorte GA, Fonseca CG, Wieloch AH, Mayer G: **Cryptic speciation in Brazilian *Epiperipatus* (Onychophora: Peripatidae) reveals an underestimated diversity among the peripatid velvet worms**. *PLoS ONE* 2011, **6**(6):e19973.

25. Whittington HB: **The enigmatic animal *Opabinia regalis*, Middle Cambrian, Burgess Shale, British Columbia**. *Philosophical Transactions of the Royal Society of London, Series B* 1975, **271**:1-43.

26. Kuhl G, Briggs DEG, Rust J: **A great-appendage arthropod with a radial mouth from the Lower Devonian Hunsruck Slate, Germany**. *Science* 2009, **323**(5915):771-773.

27. Aria C, Caron J-B: **Cephalic and limb anatomy of a new isoxyid from the Burgess Shale and the role of “stem bivalved arthropods” in the disparity of the frontalmost appendage**. *PLoS ONE* 2015, **10**(6):e0124979.

28. Liu JN, Dunlop JA: **Cambrian lobopodians: A review of recent progress in our understanding of their morphology and evolution**. *Palaeogeography Palaeoclimatology Palaeoecology* 2014, **398**:4-15.

29. Daley A, Budd G, Caron J-B: **Morphology and systematics of the anomalocaridid arthropod *Hurdia* from the Middle Cambrian of British Columbia and Utah**. *Journal of Systematic Palaeontology* 2013, **11**(7):743-787.

30. Daley AC, Edgecombe GD: **Morphology of *Anomalocaris canadensis* from the Burgess Shale**. *Journal of Paleontology* 2014, **88**(1):68-91.

31. Budd GE: **A Cambrian gilled lobopod from Greenland**. *Nature* 1993, **364**(19 August 1993):709-711.

32. Budd GE: **The morphology and phylogenetic significance of *Kerygmachela kierkegaardi* Budd (Buen Formation, Lower Cambrian, N. Greenland)**. *Transactions of the Royal Society of Edinburgh: Earth Sciences* 1999, **89**:249-290.

33. Budd GE: **Arthropod body-plan evolution in the Cambrian with an example from anomalocaridid muscle**. *Lethaia* 1998, **31**:197-210.

34. Dzik J: **The xenusian-to-anomalocaridid transition within the lobopodians**. *Bollettino della Società Paleontologica Italiana* 2011, **50**(1):65-74.

35. Martin C, Mayer G: **Neuronal tracing of oral nerves in a velvet worm—Implications for the evolution of the ecdysozoan brain**. *Frontiers in Neuroanatomy* 2014, **8**(7).

36. Cong P, Ma X, Hou X, Edgecombe GD, Strausfeld NJ: **Brain structure resolves the segmental affinity of anomalocaridid appendages**. *Nature* 2014, **513**(7519):538-542.

37. Mayer G, Martin C, de Sena Oliveira I, Franke FA, Gross V: **Latest anomalocaridid affinities challenged**. *Nature* 2014, **516**(7530):E1-E2.

38. Eriksson BJ, Stollewerk A: **The morphological and molecular processes of onychophoran brain development show unique features that are neither comparable to insects nor to chelicerates**. *Arthropod Structure & Development* 2010, **39**(6):478-490.

39. Tanaka G, Hou X, Ma X, Edgecombe GD, Strausfeld NJ: **Chelicerate neural ground pattern in a Cambrian great appendage arthropod**. *Nature* 2013, **502**(7471):364-367.

40. Haas MS, Brown SJ, Beeman RW: **Homeotic evidence for the appendicular origin of the labrum in Tribolium castaneum**. *Dev Genes Evol* 2001, **211**(2):96-102.

41. Ortega-Hernández J: **Making sense of ‘lower’ and ‘upper’ stem-group Euarthropoda, with comments on the strict use of the name Arthropoda von Siebold, 1848**. *Biological Reviews* 2016, **91**(1):255-273.

42. Budd GE: **A palaeontological solution to the arthropod head problem**. *Nature* 2002, **417**:271-275.

43. Legg DA, Caron J-B: **New Middle Cambrian bivalved arthropods from the Burgess Shale (British Columbia, Canada)**. *Palaeontology* 2014, **57**(4):691-711.

44. Legg DA, Sutton MD, Edgecombe GD, Caron J-B: **Cambrian bivalved arthropod reveals origin of arthrodization**. *Proceedings of the Royal Society B: Biological Sciences* 2012, **279**(1748):4699-4704.

45. Whittington HB: **The lobopod animal *Aysheaia pedunculata* Walcott, Middle Cambrian, Burgess Shale, British Columbia.** *Philosophical Transactions of the Royal Society of London B* 1978, **284**(1000):165-197.

46. Vinther J, Stein M, Longrich NR, Harper DAT: **A suspension-feeding anomalocarid from the Early Cambrian**. *Nature* 2014, **507**(7493):496-499.

47. Bergström Jan, Hou X-G: **Cambrian Onychophora or Xenusians**. *Zoologischer Anzeiger - A Journal of Comparative Zoology* 2001, **240**(3-4):237-245.

48. Ortega-Hernández J: **Homology of head sclerites in Burgess Shale euarthropods**. *Current Biology* 2015, **25**(12):1625-1631.

49. Ma X, Hou X, Aldridge RJ, Siveter DJ, Siveter DJ, Gabbott SE, Purnell MA, Parker AR, Edgecombe GD: **Morphology of Cambrian lobopodian eyes from the Chengjiang Lagerstätte and their evolutionary significance**. *Arthropod Structure & Development* 2012, **41**(5):495-504.

50. Scholtz G, Edgecombe GD: **The evolution of arthropod heads: reconciling morphological, developmental and palaeontological evidence**. *Development Genes and Evolution* 2006, **216**(7-8):395-415.

51. Liu J, Shu D, Han J, Zhang Z, Zhang X: **Morpho-anatomy of the lobopod *Magadictyon* cf. *haikouensis* from the Early Cambrian Chengjiang Lagerstätte, South China**. *Acta Zoologica* 2007, **88**(4):279-288.

52. Vannier J, Liu JN, Lerosey-Aubril R, Vinther J, Daley AC: **Sophisticated digestive systems in early arthropods**. *Nature Communications* 2014, **5**.

53. Dewel RA, Dewel WC: **The place of tardigrades in arthropod evolution**. *Systematic association* 1998, **Special volume 55**:109-123.

54. Ou Q, Shu D, Mayer G: **Cambrian lobopodians and extant onychophorans provide new insights into early cephalization in Panarthropoda**. *Nature Communications* 2012, **3**(1261).

55. Martin C, Mayer G: **Neuronal tracing of oral nerves in a velvet worm – Implications for the evolution of the ecdysozoan brain**. *Frontiers in Neuroanatomy* 2014, **8**.

56. Yang J, Ortega-Hernández J, Butterfield NJ, Liu Y, Boyan GS, Hou J-b, Lan T, Zhang X-g: **Fuxianhuiid ventral nerve cord and early nervous system evolution in Panarthropoda**. *Proceedings of the National Academy of Sciences* 2016, **113**(11):2988-2993.

57. Budd GE: **The morphology of *Opabinia regalis* and the reconstruction of the arthropod stem-group**. *Lethaia* 1996, **29**:1-14.

58. Budd GE, Daley AC: **The lobes and lobopods of *Opabinia regalis* from the middle Cambrian Burgess Shale**. *Lethaia* 2011, **45**(1):83-95.

59. Zhang X, Briggs DEG: **The nature and significance of the appendages of *Opabinia* from the Middle Cambrian Burgess Shale**. *Lethaia* 2007, **40**(2):161-173.

60. Haug J, Mayer G, Haug C, Briggs DEG: **A Carboniferous non-onychophoran lobopodian reveals long-term survival of a Cambrian morphotype**. *Current Biology* 2012, **22**(18):1673-1675.

61. Dzik J: **Early Cambrian lobopodian sclerites and associated fossils from Kazakhstan**. *Palaeontology* 2003, **46**(1):93-112.

62. García-Bellido DC, Edgecombe GD, Paterson JR, Ma X: **A ‘Collins’ monster’-type lobopodian from the Emu Bay Shale Konservat-Lagerstätte (Cambrian), South Australia**. *Alcheringa: An Australasian Journal of Palaeontology* 2013, **37**(4):474-478.

63. Liu J, Shu DG, Han J, Zhang Z-F, Zhang XL: **The lobopod *Onychodictyon* from the Lower Cambrian Chengjiang Lagerstatte revisited**. *Acta Palaeontologica Polonica* 2008, **53**(2):285-292.

64. Kristensen RM, Sørensen MV, Hansen JG, Zeppilli D: **A new species of *Neostygarctus* (Arthrotardigrada) from the Condor Seamount in the Azores, Northeast Atlantic**. *Marine Biodiversity* 2015, **45**(3):453-467.

65. Liu J, Shu D, Han J, Zhang Z: **Comparative study of Cambrian lobopods *Miraluolishania* and *Luolishania***. *CHINESE SCI BULL* 2008, **53**(1):87-93.

66. Van Roy P, Daley AC, Briggs DEG: **Anomalocaridid trunk limb homology revealed by a giant filter-feeder with paired flaps**. *Nature* 2015, **522**(7554):77-80.

67. Wolff C, Scholtz G: **The clonal composition of biramous and uniramous arthropod limbs**. *Proceedings of the Royal Society of London B: Biological Sciences* 2008, **275**(1638):1023-1028.

68. Hou X-G, Chen JY: **Early Cambrian arthropod-annelid intermediate sea animal, *Luolishania* gen. nov**. *Acta Palaeontologica Sinica* 1989, **28**:207-213 [In Chinese with English summary].

69. Whittington HB: **Exoskeleton, moult stage, appendage morphology, and habits of the Middle Cambrian trilobite *Olenoides serratus***. *Palaeontology* 1980, **23**(1):171-204.

70. Chen JY, Zhou GQ, Ramsköld L: **The Cambrian lobopodian *Microdictyon sinicum***. *Bulletin of the National Museum of Natural Science* 1995, **5**:1-93.

71. Briggs DEG, Fortey RA, Wills MA: **Morphological disparity in the Cambrian**. *Science* 1992, **256**(5064):1670-1673.

72. Wills MA: **Cambrian and Recent disparity: the picture from priapulids**. *Paleobiology* 1998, **24**(2):177-199.
